# The Biological Assessment and Rehabilitation of the World’s Rivers: An Overview

**DOI:** 10.3390/w13030371

**Published:** 2021-01-31

**Authors:** Maria João Feio, Robert M. Hughes, Marcos Callisto, Susan J. Nichols, Oghenekaro N. Odume, Bernardo R. Quintella, Mathias Kuemmerlen, Francisca C. Aguiar, Salomé F.P. Almeida, Perla Alonso-EguíaLis, Francis O. Arimoro, Fiona J. Dyer, Jon S. Harding, Sukhwan Jang, Philip R. Kaufmann, Samhee Lee, Jianhua Li, Diego R. Macedo, Ana Mendes, Norman Mercado-Silva, Wendy Monk, Keigo Nakamura, George G. Ndiritu, Ralph Ogden, Michael Peat, Trefor B. Reynoldson, Blanca Rios-Touma, Pedro Segurado, Adam G. Yates

**Affiliations:** 1 Department of Life Sciences, MARE-Marine and Environmental Sciences Centre, University of Coimbra, 3000-456 Coimbra, Portugal; 2 Amnis Opes Institute, Corvallis, OR 97333, USA; 3 Department of Fisheries & Wildlife, Oregon State University, Corvallis, OR 97331, USA; 4 Laboratory of Ecology of Benthos, Department of Genetic, Ecology and Evolution, Institute of Biological Sciences, Federal University of Minas Gerais, Avenida Antônio Carlos 6627, CEP 31270-901 Belo Horizonte, MG, Brazil; 5 Centre for Applied Water Science, Institute for Applied Ecology, University of Canberra, 2601 Canberra, Australia; 6 Unilever Centre for Environmental Water Quality, Institute for Water Research, Rhodes University, P.O. Box 94, Grahamstown 6140, South Africa; 7 MARE—Marine and Environmental Sciences Centre, University of Évora, 7000-812 Évora, Portugal; 8 Department of Animal Biology, Faculty of Sciences of the University of Lisbon, Campo Grande, 1749-016 Lisboa, Portugal; 9 Department of Zoology, School of Natural Sciences, Trinity Centre for the Environment, Trinity College Dublin, The University of Dublin, College Green, Dublin 2, Ireland; 10 Centro de Estudos Florestais, Instituto Superior de Agronomia, Universidade de Lisboa, Tapada da Ajuda, 1349-017 Lisboa, Portugal; 11 Department of Biology and GeoBioTec—GeoBioSciences, GeoTechnologies and GeoEngineering Research Centre, University of Aveiro, Campus de Santiago, 3810-193 Aveiro, Portugal; 12 Mexican Institute of Water Technology, Bioindicators Laboratory, Jiutepec Morelos 62550, Mexico; 13 Department of Animal and Environmental Biology (Applied Hydrobiology Unit), Federal University of Technology, P.M.B. 65 Minna, Nigeria; 14 School of Biologcal Sciences, University of Canterbury, 8140 Christchurch, New Zealand; 15 Department of Civil Engineering, Daejin University, Hoguk-ro, Pocheon-si 1007, Gyeonggi-do, Korea; 16 Pacific Ecological Systems Division, Center for Public Health and Environmental Assessment, Office of Research and Development, U.S. Environmental Protection Agency, Corvallis, OR 97333, USA; 17 Korea Institute of Civil Engineering and Building Technology (KICT), 283 Goyangdaero, Ilsanseo-gu, Goyang-si 10223, Gyeonggi-do, Korea; 18 Key Laboratory of Yangtze River Water Environment, Ministry of Education of China, Tongji University, Shanghai 200092, China; 19 Department of Geography, Geomorphology and Water Resources Laboratory, Institute of Geosciences, Federal University of Minas Gerais, Avenida Antônio Carlos 6627, CEP 31270-901 Belo Horizonte, MG, Brazil; 20 MED—Instituto Mediterrâneo para a Agricultura, Ambiente e Desenvolvimento, LabOr—Laboratório de Ornitologia, Universidade de Évora, Polo da Mitra, 7002-774 Évora, Portugal; 21 Centro de Investigación en Biodiversidad y Conservacíon, Universidad Autónoma del Estado de Morelos, Cuernavaca, 62209 Morelos, Mexico; 22 Environment and Climate Change Canada and, Canadian Rivers Institute, Faculty of Forestry and Environmental Management, University of New Brunswick, Fredericton, NB E3B 5A3, Canada; 23 Water Environment Research Group, Public Works Research Institute, 1-6 Minamihara, Tsukuba 305-8516, Japan; 24 School of Natural Resources and Environmental Studies, Karatina University, P.O. Box 1957, 10101 Karatina, Kenya; 25 Environment, Planning and Sustainable Development Directorate, 2601 Canberra, Australia; 26 Wetlands, Policy and Northern Water Use Branch, Commonwealth Environmental Water Office, 2601 Canberra, Australia; 27 Acadia University, Canada Creek, Wolfville, NS B0P 1V0, Canada; 28 Grupo de Investigación en Biodiversidad, Medio Ambiente y Salud (BIOMAS), Facultad de Ingenierías y Ciencias Aplicadas, Ingeniería Ambiental, Universidad de Las Américas, Vía Nayón S/N, 170503 Quito, Ecuador; 29 Department of Geography, Western University and Canadian Rivers Institute, London, ON N6A 5C2, Canada

**Keywords:** ecological status, freshwater, biological elements, restoration, reference conditions

## Abstract

The biological assessment of rivers i.e., their assessment through use of aquatic assemblages, integrates the effects of multiple-stressors on these systems over time and is essential to evaluate ecosystem condition and establish recovery measures. It has been undertaken in many countries since the 1990s, but not globally. And where national or multi-national monitoring networks have gathered large amounts of data, the poor water body classifications have not necessarily resulted in the rehabilitation of rivers. Thus, here we aimed to identify major gaps in the biological assessment and rehabilitation of rivers worldwide by focusing on the best examples in Asia, Europe, Oceania, and North, Central, and South America. Our study showed that it is not possible so far to draw a world map of the ecological quality of rivers. Biological assessment of rivers and streams is only implemented officially nation-wide and regularly in the European Union, Japan, Republic of Korea, South Africa, and the USA. In Australia, Canada, China, New Zealand, and Singapore it has been implemented officially at the state/province level (in some cases using common protocols) or in major catchments or even only once at the national level to define reference conditions (Australia). In other cases, biological monitoring is driven by a specific problem, impact assessments, water licenses, or the need to rehabilitate a river or a river section (as in Brazil, South Korea, China, Canada, Japan, Australia). In some countries monitoring programs have only been explored by research teams mostly at the catchment or local level (e.g., Brazil, Mexico, Chile, China, India, Malaysia, Thailand, Vietnam) or implemented by citizen science groups (e.g., Southern Africa, Gambia, East Africa, Australia, Brazil, Canada). The existing large-extent assessments show a striking loss of biodiversity in the last 2–3 decades in Japanese and New Zealand rivers (e.g., 42% and 70% of fish species threatened or endangered, respectively). A poor condition (below Good condition) exists in 25% of South Korean rivers, half of the European water bodies, and 44% of USA rivers, while in Australia 30% of the reaches sampled were significantly impaired in 2006. Regarding river rehabilitation, the greatest implementation has occurred in North America, Australia, Northern Europe, Japan, Singapore, and the Republic of Korea. Most rehabilitation measures have been related to improving water quality and river connectivity for fish or the improvement of riparian vegetation. The limited extent of most rehabilitation measures (i.e., not considering the entire catchment) often constrains the improvement of biological condition. Yet, many rehabilitation projects also lack pre-and/or post-monitoring of ecological condition, which prevents assessing the success and shortcomings of the recovery measures. Economic constraints are the most cited limitation for implementing monitoring programs and rehabilitation actions, followed by technical limitations, limited knowledge of the fauna and flora and their life-history traits (especially in Africa, South America and Mexico), and poor awareness by decision-makers. On the other hand, citizen involvement is recognized as key to the success and sustainability of rehabilitation projects. Thus, establishing rehabilitation needs, defining clear goals, tracking progress towards achieving them, and involving local populations and stakeholders are key recommendations for rehabilitation projects (Table 1). Large-extent and long-term monitoring programs are also essential to provide a realistic overview of the condition of rivers worldwide. Soon, the use of DNA biological samples and eDNA to investigate aquatic diversity could contribute to reducing costs and thus increase monitoring efforts and a more complete assessment of biodiversity. Finally, we propose developing transcontinental teams to elaborate and improve technical guidelines for implementing biological monitoring programs and river rehabilitation and establishing common financial and technical frameworks for managing international catchments. We also recommend providing such expert teams through the United Nations Environment Program to aid the extension of biomonitoring, bioassessment, and river rehabilitation knowledge globally.

## 1. Introduction

Since the beginning of human civilization, we have used, exploited, and degraded freshwater ecosystems, beginning with agriculture in the fertile lands around rivers, then by industrialization, and in the second half of the 20th century by urbanization [1–3]. The consequences are manifold, from water pollution to highly modified channels, riparian zones, and flow regimes [4–6]. This has resulted in catastrophic and accelerated loss of freshwater biodiversity, increased prevalence of invasive non-native species, and altered ecosystem functioning [4,7].

Thus, the necessity of assessing the ecological condition of rivers arose, first to prevent public health problems and more recently to understand the extent of damage in the ecosystems and plan cost-effective recovery actions [8,9]. Ecological assessments assume that rivers should be viewed as ecosystems, composed of different biological assemblages that interact among themselves and with the abiotic conditions, all contributing to complex ecosystem functions. The biological elements integrate the alterations and pollution of the ecosystem over time and space and are therefore good indicators of river ecological condition [9,10].

The first biotic indices for assessing rivers appeared in the middle of the 20th century [10]. Initially, they were based on the microbial effects of organic pollution [11], leading to the development of saprobic indices. Later, indices were developed for diatoms and macroinvertebrates [12,13]. For fish, one of the first widely implemented approaches was the Index of Biotic Integrity (IBI) that responded to the goals of the Water Pollution Control Act of 1972 in the USA [14,15]. The use of biological assemblages (i.e., benthic invertebrates, fish, algae, or other plants) as indicators was integrated into other pieces of legislation such as the Resource Management Act (1991) in New Zealand, the European Water Framework Directive (WFD; [16]), or CONAMA—Conselho Nacional do Meio Ambiente 357/2005 in Brazil. However, despite the large number of existing methods [10,17], the extent to which the biological assessments of rivers and streams are performed worldwide is unclear, as information is largely scattered.

Theoretically, the assessment of river or stream conditions is just an intermediate step that could lead to rehabilitating rivers that fail to meet the quality criteria. Yet, how much has been done to improve the quality of rivers at a global scale is even more obscure. In addition, restoration or rehabilitation of rivers may happen for different number reasons, often independent of their ecological condition. Common reasons for rehabilitation are the will to improve the aesthetics of an urban area near a river, implement ecological flows to comply with existent legislation, prevent floods, or facilitate fish passage at barriers. Here we use the word rehabilitation instead of restoration because nearly all recovery efforts focus on rehabilitating river ecosystem services for humans in small river reaches—not restoring entire river ecosystems to some pristine or natural state [18].

Regardless of the reasons for rehabilitation projects, often there is no associated ecosystem monitoring, and therefore the amelioration of ecological quality is unknown. This prevents learning about the most effective measures to restore rivers. In addition, the ideal measures may not be feasible because of high costs that may be a barrier to implementation. Additionally, different nations sharing the same catchments may differ widely in the degree to which they protect the environment and use natural resources, which hinders continental and global improvement. Yet, the aims of the United Nations Agenda 2030 for sustainable development clearly state the need to decrease pollution and guarantee access to safe drinking water for all and protect the freshwater aquatic ecosystems and biodiversity.

Thus, the rehabilitation of rivers is a global challenge and one that we must overcome if we aim to achieve global sustainability/water security. To understand the status of rivers biological assessment and rehabilitation, we examine for the first time together, the major programs and studies in Africa, Asia, Europe, Oceania and North, Central and South America to determine: (i) where and how (i.e., which methods, legal basis), biological assessment is used; (ii) the drivers for river rehabilitation and assessment; and (iii) the essential attributes that facilitate successful rehabilitation programs.

**Figure 1.**
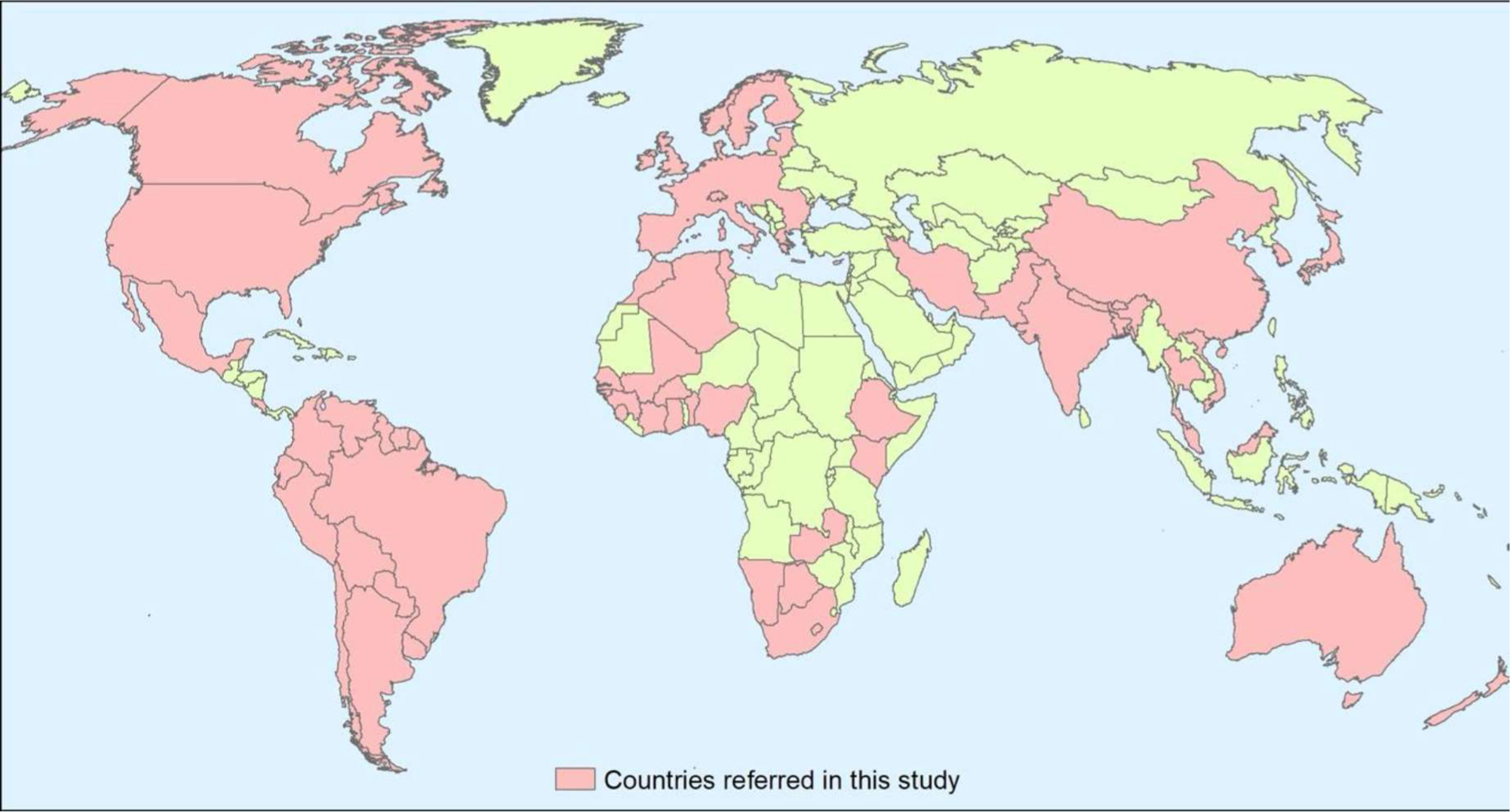
Countries referred in this study regarding their status in biological monitoring and rehabilitation of rivers.

We then identify major gaps and opportunities for research and targets for future investment. The results of such analysis should enable the establishment of international directions towards the protection and recovery of freshwater ecosystems. Although we aimed to give an overview of the globe, data were only collected in the 82 countries indicated in Figure 1 and for which relevant information is available—Supplementary Table S1 summarizes the main monitoring programs gathered around the world and Supplementary Table S2 addresses relevant river rehabilitation projects in the 5 continents.

## 2. Biological Assessment of Rivers

### 2.1. Africa

The implementation and uses of bioassessment programs are diverse across the African continent. The ways bioassessment is used in Africa can be grouped into four broad categories: (i) setting management targets for rivers and monitoring for their compliance; (ii) monitoring the ecological conditions of freshwater ecosystems; (iii) monitoring in response to specific event/projects; and (iv) as part of water use license conditions. However, there are substantial overlaps between these bioassessments (Table S1).

In South Africa, instream targets known as the resource quality objectives (RQOs) are set for every water body [19,20]. RQOs are qualitative and quantitative descriptions of the physical, biological, and chemical attributes of a water body at the appropriate level of protection, after concurrent consideration of requirements for ecosystem functioning and socio-economic imperatives. Objectives are set for fish, riparian vegetation, and macroinvertebrate assemblages against which monitoring results are then interpreted for compliance. These objectives are mandated in the National Water Act of South Africa (Act No. 36 of 1998). Reference conditions for biological elements were set based on historical data sets, expert judgment, and data from minimally disturbed sites [21].

The River Ecostatus Monitoring Programme (REMP) is the official national bioassessment program in South Africa [22]. REMP is designed to generate ecological data to support the management of rivers and streams. Several formalized protocols, tools, and indices for the biological elements have also been developed [21,23]. The fish, riparian vegetation, and macroinvertebrate assessment indices (the FRAI–[23], VEGRAI–[24], and MIRAI–[25]; respectively) are used for the routine ecological monitoring of rivers and streams. Each of these indices is composed of metrics, integrating biotic responses/preferences to alteration in the physical habitat, water quality, depth, shade/cover, and flow velocity. Results obtained through the indices are interpreted as the degree of deviations to reference condition, where A indicates no deviation/natural condition and F indicates the greatest deviation.

Elsewhere within Southern Africa, protocols for ecological assessment have been developed based on macroinvertebrate assemblages: the South African Scoring System (SASS), the Zambian Scoring System (ZISS) [26], the Namibian Scoring System (NASS) [27], and the Okavango Assessment System (OKAS) [28,29]. ZSS, NASS, and OKAS are modifications of SASS. SASS was originally modified from the BMWP (Biological Monitoring Working Party) and is presently in version 5 (SASS5) [30]. The results of these indices are expressed both as an index score (e.g., SASS5 score) and as an average score per recorded taxon (ASPT). There are also assessment protocols based on fish such as the Fish Assemblage Integrity Index (FAII) developed and used within the region [31].

In West Africa, recent studies have been directed towards establishing reference conditions for minimally disturbed sites using algae, macrophytes, benthic macroinvertebrates, and fish, which provide the foundation for bioassessment within the Sahel region [32]. Multimetric indices (MMI) based on macroinvertebrates-based were developed for the Zio River Basin (MMIZB; [33]) and to assess urban rivers within the Niger Delta region [34]. In Eastern and Central Africa, several biological assessment studies have been carried out mainly for scientific purposes. Indices of Biological Integrity (IBI) have been used to describe biological conditions in rivers based on macroinvertebrates (e.g., [35–44], diatoms [45–47], and fish [48]).

Other authors have explored trait-based approaches in Africa. Odume [49] identified signature traits to assess urban pollution in South Africa and a trait-based approach was useful for exploring the effects of fine sediments on riverine ecosystems [50,51]. In West Africa, Edegbene et al. [52] explored the pattern of distribution of traits and ecological preferences to urban and agricultural pollution. However, the greatest challenge is the paucity of life-history information on Afro-tropical macroinvertebrates and only one trait database has been compiled in the region [53].

The existing large-extent bioassessment programs in Africa have been triggered in response to specific events. The best-known example is the Onchocerciasis control program set up in response to the use of insecticides for controlling river blindness in West Africa. To evaluate the possible short- and long-term effects of insecticide application on non-target fauna, a biomonitoring program was set up between 1974 and 2003 covering 50,000 km of river and including 11 West African countries. The biomonitoring results demonstrated little effect on fish [54] and macroinvertebrate taxonomic and functional composition [55,56].

Most dischargers into a receiving stream or river are required to undertake their bioassessment, up-and down-stream of the effluent discharge point. For this purpose, macroinvertebrate assemblages using the SASS5 protocol have been used, as well as other biological assemblages such as fish and riparian vegetation, depending on the nature of the water use.

A diversity of stakeholders also implement bioassessments in Africa, and their programs include transboundary (regional), national, and local extents. The actors or stakeholders may be: (i) transboundary river commissions, international funding/multilateral institutions, (ii) national governments, (iii) community/citizens, and (iv) non-governmental organizations. For example, the Orange-Senqu River Commission (ORASECOM) is mandated to ensure equitable and sustainable management of water resources in the transboundary Orange-Senqu River basin, which covers 1,000,000 km^2^. The river basin is located in four nations: South Africa (64.2% of its total coverage), Namibia, Botswana, and Lesotho. To date, ORASECOM has implemented two basin-wide bioassessment surveys using macroinvertebrates, fish, and riparian vegetation [57] by using common protocols developed in South Africa.

Recently, the Nairobi Water Trust Fund in Kenya began supporting a pilot study to develop and use the USEPA’s Biological Condition Gradient (BCG) approach [58] in the Upper Tana River watershed in Kenya, to assess water and landscape conditions to inform rehabilitation initiatives. In Lesotho, the Lesotho Highland Development Authority regularly monitors waters through macroinvertebrates, fish and riparian vegetation.

Various community-based monitoring initiatives have also been in place for implementing bioassessment programs. In Gambia and Ghana bioassessment is currently being accomplished through community-based monitoring [59,60]. In Southern Africa, community-based monitoring is widespread. A formal community-based assessment protocol called Mini-SASS has been developed using macroinvertebrates [61]. MiniSASS (http://www.minisass.org/en/) requires minimal training and citizens can collect and upload data into an online platform, using the MiniSASS app. Mini-SASS is also implemented in East Africa through a citizen science initiative called ‘Adopt a River’ in which students in primary and high schools assess river ecological conditions by measuring water physico-chemical variables and macroinvertebrates.

In North Africa, despite the absence of established bioassessment programs, research and short-term assessments have been undertaken in rivers, namely in Morocco, Algeria and Tunisia. In Morocco, standardized methodology and well-known indices have been used such as: the IBD (Indice Biologique Diatomées) based on diatoms [62,63] (Fawzi et al.); the IBMR (Indice Biologique Macrophytes Rivières) for macrophytes [64] Bentaibi et al., 2014); the IBMWP (Iberian BMWP) [65–67] and the IBGN (Indice Biologique Global Normalisé) [68,69] for macroinvertebrates; and the QBR (Index of Riparian Quality) for riparian vegetation [67]. Other studies have adapted the principles of the European WFD to Maroc-can rivers [66,70,71]. In Algeria, freshwaters have been recently assessed with benthic invertebrates to detect the effects of pollution through use of indices and metrics such as the BMWP, IBGN and EPT in Kebir-Rhumel or Guebli river catchments and streams in the Belezma National Park (Biosphere Reserve) [72–74]. Diatoms have also been used to obtain quality assessments of rivers in Algeria using the BDI index [75] and in Tunisia through diatom diversity in Lake Ischkeul [76].

### 2.2. Asia

Biological monitoring of rivers is not a common approach in the majority of Asian countries, instead, monitoring of freshwater quality is primarily undertaken via traditional physiochemical and microbial measurements. However, it is applied in Japan, South Korea, Singapore and China to different degrees ([77–79]; Table S1).

In Japan, the Ministry of Land, Infrastructure, Transport and Tourism (MLIT), having responsibility for 109 major Japanese river systems, launched the Nature-oriented river works in the 1990s [80]. At the same time, MLIT initiated the National Census on the River Environment (NCRE) to gather nation-wide baseline information on the ecological state of river corridors. Following standardized protocols, data on fish, benthic invertebrates, plants, birds, terrestrial insects, amphibians, reptiles and mammals are collected from 109 Class A and 133 Class B rivers at five (or ten)-year intervals. All data are checked through a rigorous peer-review process and made publicly available in a river environmental database (http://www.nilim.go.jp/lab/fbg/ksnkankyo/). This regular monitoring not only provides important information on long-term trends in biodiversity but also improves scientific understanding of river ecosystems [81]. It provides important baseline information to support River Improvement Plans (basic plans for river management for 20–30 years) and to identify priorities for river rehabilitation. The long-term NCRE results (ca. 30 years) indicate a decline in riparian biodiversity, invasion of non-native species and a decline in the number of spawning bitterlings [82]. However, the lack of reference sites and ecological quality goals are considered drawbacks when compared with other monitoring programs.

The limited number and area of NCRE survey points for each river, however, preclude planning reach-extent rehabilitation projects and having a catchment-scale biodiversity perspective. Therefore, the MLIT began a pilot-study in 2019 using environmental DNA to investigate fish diversity with the goal of costs reduction and increased number of monitoring sites. Additionally, Airborne Lidar bathymetry (ALB) has been used for river surveys and allowed to facilitate three-dimensional habitat distribution and watershed-scale ecological niche modelling.

In the Republic of Korea, guidelines for aquatic ecology surveys are in operation to evaluate river rehabilitation projects. The legal basis for these guidelines is governed by the Water Environment Conservation Act. The Enforcement Rules of this Act stipulate surveys of aquatic ecosystem health. South Korea’s National Aquatic Ecological Monitoring Program (NAEMP) began research in 2003 and was implemented on 540 to 800 sites annually. In 2009, its results indicated that 2%, 9%, 25% and 25% of sites scored as poor based on riparian, macroinvertebrate, fish and diatom MMIs, respectively. The Water Quality and Aquatic Ecosystem Conservation Act stipulated a survey on ecological health, covering over 3000 sites.

The Guidelines for the Survey of Aquatic Ecology and Methods for Health Assessment stipulate assessing diatoms (Trophic Diatom Index), benthic macroinvertebrates (Benthic Macroinvertebrate Index) and riparian vegetation (Riparian Vegetation Index). All three indices include species composition and other metrics such as: diatom abundance per species; number of macroinvertebrate endemic, sensitive, tolerant, abnormal and hybrid species; and vegetation cover, area of annual plants, presence of non-native species, species evenness of hygrophytic plants and number of tolerant species for riparian vegetation. The reference values of the indices are adjusted to the natural characteristics of Korea’s rivers and past monitoring results and research.

The Singapore Public Utilities Board conducts regular water quality monitoring across canals, rivers and reservoirs [78]. The use of biotic indices in national ecological monitoring programmes to detect pollution began in October 2011. Benthic invertebrates have been sampled in 47 streams and 17 reservoirs and quarry lakes. Two benthic invertebrate pollution indices have been developed primarily to assess metal pollution [78,79]. No other biological parameters (such as algae, functional indicators or fish) are measured.

In China, the Ministry of Water Resources issued in 2015 the “Guidelines for Aquatic Ecological Protection and Restoration Planning”. This requires monitoring fish, aquatic mammals, benthic invertebrates, epiphytic algae, phytoplankton, aquatic vascular plants, waterside vegetation, beach vegetation, amphibians, reptiles, wetland birds, and rare, endangered and endemic species. The assessment indices include predictive models and multi-metric indices but the latest is more widely used in China for both diatoms and macroinvertebrates (e.g., [83,84]). Yet, up to now China has not conducted a national river ecological survey.

From 2000 until now, a total of 122 large and small Chinese rivers were assessed through national projects (e.g., National High-Tech R&D Program, National Water Pollution Control and Treatment Science and Technology Major Project, National Key Basic Research and Development Project). Between 2008 and 2020, a long-term monitoring project was undertaken and the biological indicators included: algae (number of species, algae cell density, Shannon–Weiner Diversity Index, index of biotic integrity), macroinvertebrates (total number of taxa, EPT-Ephemeroptera, Plecoptera, and Trichoptera, BMWP), and fish (fish species abundance, Shannon Winer diversity, density, IBI). There are also catchment/province-wide monitoring programs, such as that in the Yangtze River (launched in 2005, by the Yangtze River Water Resources Commission), and the Yellow River. According to the latest 2020 Yangtze River Vitality Report, the water ecology of the Yangtze River is grade B-, which means unhealthy. The ecological quality in the middle and lower reaches is poor. The existing monitoring programs have shown a serious degradation of aquatic ecosystems in several important Chinese watersheds. Xing et al. [85] reported that 70 and 85 endemic Chinese fish species are threatened or endangered, respectively; and the endemic Yangtze River dolphin, Chinese sturgeon and Chinese paddlefish are functionally extinct [86].

The national implementation of river and lake monitoring would boost the application of biological elements in other Chinese hydrographic basins. Yet, there is still lack of evaluation and practical cases that reflect the regional characteristics and intercalibration exercises are needed. A pilot cooperation project supported by the European Union and Peoples’ Republic of China is being developed in an attempt to adapt the WFD monitoring approach and Chinese guidelines and contribute to the design of monitoring and rehabilitation plans in China with case studies in the Haihe, Taihu and Nanxi basins.

In other Asian countries, several studies have also been undertaken to investigate the use of biological indicators in the quality assessment of rivers. Using benthic invertebrates, several examples come from India, including in the Kailash Mountains of Uttarakhand state [87] and Kerala region [88,89]. Various biotic indices were tested including SIGNAL [90] and BMWP. In Thailand, the BMWP was also applied to samples from the River Ping [91]. In addition, a new biotic index (HKHbios, Hindu Kush-Himalayan biotic score) was proposed [92] for the Hindu Kush-Himalaya region that crosses 5 countries (Bangladesh, Bhutan, India, Nepal and Pakistan). In Vietnam a MMI was developed for the Cau river basin [93] and a similar approach was used in Iran [94]. Diatom indices such as the TDI and IPS have been tested in the Chambal River, Central India [95] and in the Kathmandu Valley streams of Nepal and India [96]. In India, the IBI was also applied for assessing the fish assemblage condition of the Khan and Kshipra Rivers [97] and fish species diversity was assessed in the western Ghats [98–100].

### 2.3. Central and South America

In South America, bioassessment is not homogeneous: there are countries with well-structured and consolidated monitoring programs based on chemical, physical and microbiological parameters (e.g., Brazil, Peru, and Uruguay); others have well-defined legal guidelines but no national initiative (e.g., [101–104]); and others with no prospect for monitoring (e.g., Venezuela, Suriname, Guiana). However, there is no official national program that uses biological indicators in any South American nation (Table S1).

In some countries, environmental impact assessment includes aquatic biota, with macroinvertebrates and fish being the most common (e.g., Ecuadorian normative for Mining Impact assessment). In a few cases, legislation on the use of biological indicators to assess rivers exists but is not applied by governments. One example is Costa Rica, which legislated the classification and evaluation of surface waters based on aquatic macroinvertebrates [105] and very recently the streams and riparian areas protection and conservation (Política Nacional de Áreas de Protección de Ríos Quebradas, Arroyos y Nacientes 2020). In Brazil, CONAMA Resolution 357 [106] focused on waters for human uses and supports the use of aquatic assemblages or organisms to assess water bodies and the importance of maintaining riparian vegetation to protect aquatic biodiversity and preserve ecosystem services. In the Brazilian state of Minas Gerais, Joint Normative Resolution COPAM/CERH-MG No. 1 [107], establishes that aquatic environments should be assessed by biological indicators, with the determination of reference areas, assessment methods and bioindicator groups, including fish and benthic invertebrates. Colombia also has guidelines for implementing standardized ecological monitoring of aquatic assemblages, in addition to an ecological quality index [108].

Nevertheless, in the last 20 years a considerable amount of information has been produced in South and Central America on the ecology, biodiversity and response of organisms to environmental stressors in rivers [108] such as in: Uruguay [109]; Paraguay [110]; Bolivia [111]; Chile [112]; Colombia [113]; Peru [114]; Ecuador [115]; Guiana Francesa [116]; Venezuela [117]; Brazil [118,119]. Most studies were conducted based on a single or few temporal samplings with some exceptions covering multiple years, such as in studies of the Rio das Velhas basin, Minas Gerais, Brazil [120,121].

Several countries follow the reference condition approach (e.g., [111,114]—Bolivia; [115]—Ecuador and Perú, [122]—Brazil; [112]—Chile). In the Brazilian neotropical savanna and Atlantic Forest biomes, the establishment of reference conditions for ecoregions have also been investigated [123,124], respectively. Additionally, over time a variety of biological indices have been applied and adapted for macroinvertebrate assemblages: BMWP ([125]—Brazil; [113]—Colombia; [126]—Bolivia, [115]—Ecuador and Peru]; taxonomic richness [122,127], EPT richness [128] and Saprobiotic index [129]. However, over the past decade, there has been a convergence on the use of MMIs using various characteristics of macroinvertebrate and fish assemblages (e.g., [122,130–133]), and predictive models [111,134,135]. Some studies also follow trait-based approaches for assessing neotropical rivers (e.g., [135–141].

Non-native and invasive species have also been used as bioindicators in the reservoirs of hydroelectric dams in large river basins of South America, like the Parana [142] and São Francisco [143,144]. Despite the existence of specific legislation dealing with non-native and invasive species by the Brazilian Environment Minister (CONABIO 07), there have been no ecological assessments dedicated to them.

Some recent studies incorporated probabilistic sampling, threshold analyses and citizen monitoring. Probabilistic study designs have allowed expansion of site results to entire catchments or hydrologic units (e.g., [122,145,146]). Finally, public schools have also implemented biomonitoring of urban streams (e.g., [147]).

Despite all this knowledge, political and financial constraints remain serious obstacles to implementing official programs, instead, existing programs have been operated by individuals at research institutions. In Brazil, universities have been at the forefront of monitoring processes using the standardized methodologies adapted from the US Environmental Protection Agency, with studies conducted in various biomes including Amazonia [130,137], Cerrado [89], Atlantic Forest [145,148], and Caatinga [149]. Also, in 2008 the state of Minas Gerais (Brazil) took the initiative to use biological indexes through technical state studies, but the objective was never consolidated as public policy. In Argentina, each province has its own regulations for water quality classification, but recently scientists from across the country launched the standardization of procedures, class boundaries, and indices. In Ecuador, in the frame of the National Action Plan for Biodiversity (2015–2020), a series of aquatic indicators were proposed for monitoring aquatic ecosystems [150]; however, they were never implemented. Recently, a network of Ibero-American researchers has compiled different methodologies for effective biomonitoring with the aim of an inter-calibration among countries (http://www.ibepecor.csic.es/en/, [151]).

### 2.4. Europe

In the European Union (EU), the Water Framework Directive (WFD) [16] is an umbrella for establishing ecological assessment programs in its 27 member-states. The WFD considers water a patrimony that should be protected for future generations and that all European water bodies should be recovered to Good ecological status (now by 2027). To achieve that aim it proposes a complex plan that includes catchment characterization, establishing water body types and delineating programs of measures to protect and recover the ecological status of water bodies.

The novelty of the WFD compared to previous legislation is that ecological assessment focuses primarily on the conservation of aquatic assemblages whereas water physico-chemical characteristics and hydromorphology are considered supporting elements. In rivers the compulsory biological quality elements are benthic macroinvertebrates, fish and aquatic plants, usually divided into the diatoms (microalgae) and macrophytes. Finally, natural water bodies must be classified into 1 of 5 Ecological Quality Status (EQS) classes: High, Good, Moderate, Poor or Bad. This status (or ecological potential in the case of artificial or heavily modified water bodies) is obtained through the combined classifications of biological elements with water chemistry and hydromorphology (usually based on the one-out all-out approach). Good status is the minimum acceptable quality that does not require measures for its improvement and where the biological elements deviate only slightly from near-natural reference conditions [152].

Before the implementation of the WFD, a diversity of indices existed such as: the BMWP and IBMWP, ASPT, BBI (Belgium Biotic Index) [153–155] or predictive models (e.g., [156,157]) based on macroinvertebrates; the TDI (Trophic Diatom Index), IPS (Indice de Polluosensibilité Spécifique), CEE (European Economic Community Index), IBD, GDI (Generic Diatom Index) for diatoms [158–160]; and the IBI and derived indices for fish [14–16]. Macrophytes were assessed with the IBMR, MTR (Mean Trophic Rank) and RVI (Riparian Vegetation Index) [161–164].

After the WFD, the index values had to correspond to deviations from reference conditions that were established for each river type and that needed to be comparable across the EU [165,166]. Sampling and analytical methods had to be compliant with guidelines such as, addressing the composition and abundance of biological elements, fish age structure, and quality classes that correspond to similar disturbance levels. This generated an exponential development of new methods for the biological assessment of rivers [17,167,168]. Finally, the assessment methods had to go through an Intercalibration Exercise (IC) that aimed to guarantee the comparability of classifications among MS [149,167,169–173]. This exercise was undertaken in groups of countries with similar types of rivers, the Geographic Intercalibration Groups (GIGs), such as the Northern, Alpine, Central Baltic, Eastern Continental and Mediterranean [167,171,174].

As a result of the IC process, many official methods adopted for macroinvertebrates by member states became structurally similar [175,176]. Most countries adopted a multimetric approach, such as Italy (STAR_ICMi—Intercalibration Common Metric index), Portugal (IPtI—Indice Português de Invertebrados), Spain (IMMi—Iberian Mediterranean Multimetric index), Ireland (MacrOper), Germany (Perlodes), Belgium (MMIF—Multimetric Macroinvertebrate Index Flanders), Austria, Sweden (DJ index) [6,166,177–179]. Yet, other approaches still coexist such as the biotic indices IBGN for France and the Iberian BMWP in Spain, and predictive models (RIVPACS—River Invertebrate Prediction and Classification System /RICT—River Invertebrates Classification Tool) in the UK [176].

In addition to taxonomic-based indices, the use of macroinvertebrate biological traits in the assessment of rivers has been investigated by several European authors as a way of assessing river ecological functioning (e.g., [10,180–182]). One example of an index integrating traits is the I2M2 index [183] developed in France, which includes metrics such as the relative abundance of polyvoltine taxa or the relative abundance of ovoviviparous taxa. However, the use of traits has not yet been formally adopted by EU countries.

For diatoms, most methods adopted by member states are based on the autoecology of species (i.e., indicator value and sensibility to degradation) translated into diatom indices. Some countries adopted the IPS (Coste in Cemagref; [184]), such as Portugal, Cyprus, Sweden, Bulgaria among others [172,176]. In Spain, the MDIAT diatom method was adopted for northern rivers [185] whereas France adopted the IBD [186] and Italy the Intercalibration Common Metric Index (ICMi—[187]). Slovenia’s method is based on Rott’s Saprobic and Trophic index [188,189]. In the UK, the assessments of a new tool called Diatoms for Assessing River and Lake Ecological Quality—DARLEQ2 is combined with macrophyte assessments (LEAFPACS 2) to give an overall classification for aquatic flora (macrophytes and phytobenthos).

At the publication date of the WFD, there was almost no tradition in the assessment of water quality in Europe using macrophytes, despite pioneer biological and ecological studies on aquatic plants (e.g., [190,191]). Historically, acquiring field data on aquatic plants was challenging concerning taxonomic capability and to the practical implementation of surveys [192]. However, the knowledge that macrophyte species could be consistently found across biogeographical regions and were related to water quality led to the development of several indices [170]. The initial pool of metrics originally addressed nutrient enrichment and was based on the bioindicator performance of macrophytes along a degradation gradient from reference (oligotrophic) to highly impaired river systems [193]. However, many of these national efforts were unsuccessful because of the lack of correlation with pressures, lack of reference standards or failure in the harmonization procedure (e.g., sampling and taxonomic precision) [167]. Presently, only 15 official metrics are used after the WFD inter-calibration exercise [17,170]. Besides eutrophication, macrophytes also respond to general degradation, which includes multi-pressures such as organic matter, hydromorphological alterations or single nutrients.

The fish national methods included in the IC [173,194] ranged from MMIs (most countries), to predictive models, as is the case of the methods adopted by England-Wales, Ireland and Scotland. The French method is a guild-based metric, although also relying on predictive models of species occurrence [195]. The Portuguese index for fish, F-IBIP (Índice Piscícola de Integridade Biótica para Rios Vadeáveis de Portugal Continental, [196]), is a typical type-specific MMI for wadeable streams based on the reference condition approach. Metrics are either based on presence/absence or abundance data and are included in six main functional attributes: composition, general tolerance, trophic function, habitat preference, reproductive classification, potamodromy and age structure [173]. In the Intercalibration Exercise, a common pan-European fish index, the EFI+ index was developed (European Fish Index, [197]). This is a multimetric guild-based index that uses a predictive model to derive expected taxa under reference conditions from abiotic environmental characteristics of individual sites and quantifies the deviations between the observed and predicted fish assemblages [173].

The monitoring guidelines of the WFD do not explicitly take into account non-native species [198]. However, the presence of non-native invertebrates, particularly when invasive, has influenced the results of some indices for benthic macroinvertebrates, including those used for the WFD assessment [199]. Also, the detection of rare species is generally beyond the scope of WFD monitoring. In fact, the design of the monitoring procedures emphasizes time and cost efficiency to maximize the number of sites to be sampled, which often means the production of taxonomic lists at a coarser taxonomic resolution than species (e.g., families for macroinvertebrates). Some studies have assessed this trade-off as acceptable [200], but others have identified an impact on the assignment of EQS [199,201].

The monitoring networks established in the EU aim to generate comprehensive and coherent information on the ecological status of all waterbodies, across all river basin districts, providing crucial data for elaborating River Basin Management Plans. There are three types of monitoring each with different objectives. (1) Surveillance monitoring assesses natural and anthropogenic long-term changes in river basins [202]. It includes biological, hydromorphological, and general physico-chemical quality parameters, as well as pollutants. (2) Operational monitoring assesses possible changes through time in the status of those water bodies for which a reasonable concern of not meeting environmental objectives exists. The quality elements to be assessed depending on the relevant pressures. (3) Investigative monitoring is conducted when a water body is not meeting environmental objectives because of an unknown cause or a pollution spill. The last two types of monitoring determine measures to achieve environmental objectives.

In addition to WFD ecological monitoring, the EU Habitats Directive requires member states to monitor selected habitats and species (Annexes IV and V), both in and outside of Natura 2000 sites. These lists include several taxonomic groups from the freshwater realm: odonates, fish, gastropods and bivalves.

The nation-wide monitoring programs of member states serve for elaborating River Basin Management Plans (RBMP). The WISE Water Framework Directive database (https://www.eea.europa.eu/data-and-maps/data/wise-wfd-4) has been gathering data from the 1st and 2nd River Basin Management Plans reported by EU members states, Norway and the United Kingdom. In the 2nd RBMP the member states implemented a surveillance monitoring of 19,637 river sites and operational monitoring in 67,691 sites [203]. Based on information communicated by member states, for 77% of the European rivers, 62% are predicted to fail the minimum quality of Good status. The major pressures affecting the European rivers are pollution and hydromorphological alterations, namely longitudinal barriers and alteration or removal of riparian vegetation [204–206]. The major driver of failure to achieve Good chemical status is excess nutrients (phosphorous and nitrogen) and pesticides. Around 7% of the European surface water bodies (not only rivers but also lakes) are affected by significant water abstraction, especially in southern Europe.

### 2.5. North America

The policy foundation for USA lotic ecosystem monitoring and assessment is the Federal Water Pollution Control Act (Clean Water Act; CWA, 1972). The objective of the CWA “is to restore and maintain the chemical, physical, and biological integrity of the Nation’s waters.” More specifically it aims to “provide for the protection and propagation of fish, shellfish, and wildlife and recreation in and on the water.” Each state must provide a biennial report on the water quality of all navigable waters, including the extent to which they meet the objectives and goals of the CWA, and including “the nature and extent of nonpoint sources of pollutants.”

Because they lacked standard monitoring, analysis and reporting protocols, those State reports could not be used to assess the ecological status or trends in all USA waters [207]. As a result, USEPA initiated the Environmental Monitoring and Assessment Program (EMAP), which initially focused on a set of methodological studies (e.g., [208–210]). Those studies were followed by regional demonstration projects (e.g., [211–213]). The demonstration projects led to a wadeable stream assessment (WSA) across the conterminous USA [214,215]. The success of the WSA led to the establishment of the National Rivers and Streams Assessment (NRSA; [58]). NRSA surveys were conducted in 2008–2009, 2013–2014, and 2018–2019 during the summer base flow period through use of standard protocols [216,217]. To date, nearly 5000 sites have been sampled, over 1000 of which have been resampled to refine trend and sampling error assessments.

The NRSA, like all NARS programs, is based on a probability (dispersed, randomized) survey design for site selection [218,219] and a systematic site sampling design [220]. At each wadeable stream and boatable river site, a standard suite of biological, physical, and chemical condition indicators is sampled. The raw physical habitat data are converted into many quantitative metrics [221], including measures of habitat condition, relative bed stability [222,223] and hydrologic retention [224]. Biological elements include fish, macroinvertebrate and periphytic diatom assemblages. The accuracy and precision of fish field and macroinvertebrate and diatom taxonomic identifications have been assessed and improved in independent studies [225–228]. The raw biological data are converted into indices (MMI, observed/expected models) for reporting at state, ecoregional and national spatial extents [213,215,229]. Ecological assessments are based on ecoregional reference site conditions [230,231] and models [232]. The macroinvertebrate metrics include taxonomic composition, diversity and richness, as well as feeding, habit, and tolerance guilds. Fish MMIs are based on trophic, habitat, reproductive, life history and tolerance guilds, as well as taxonomic composition and non-native species. The physical and chemical indicators are used in conducting biological risk assessments [233]. Because all sites are sampled with standard methods and randomly selected, the results can be inferred to all target waters in the conterminous USA, which are important strengths of the NRSA. The NRSA data are publicly available at https://www.epa.gov/national-aquatic-resource-surveys/what-national-rivers-and-streams-assessment#tab-2.

The NRSA has produced 11 key findings [216,217,229]. (1) The biological condition of conterminous USA stream and river length is 30% good, 28% fair, and 44% poor based on macroinvertebrate multimetric index (MMI) scores. (2) This varies regionally, with 64% and 26% in poor condition in the Coastal Plains and the Southern Plains, respectively. (3) For fish assemblages, stream and river length is 26% good, 22% fair, and 37% poor, varying regionally with 60% and 24% poor in the Coastal Plains and Northern Plains, respectively. Nationally, 14% of the stream and river length (ranging from 5–35% regionally) could not be assessed for fish assemblages because of the likely presence of threatened or endangered species. (4) Poor macroinvertebrate condition is nearly twice as likely in USA rivers and streams with high acidification, phosphorus, nitrogen, salinity and sedimentation levels, or low riparian vegetation cove. (5) In the West, excessive levels of nitrogen, sedimentation, and salinity were 3–4 times as likely to lead to poor macroinvertebrate condition. (6) The environmental predictors of biological metrics, and the strengths of their relationships, varied among ecoregions and between fish and macroinvertebrate assemblages. (7) Anthropogenic predictors were more important for explaining metric scores than natural variables. (8) This was also true of site-extent predictors versus catchment-extent predictors. (9) Fish and macroinvertebrate indices were weakly correlated, and (10) differed in the strength of their responses to different sets of predictors. (11) The above results support using multiple assemblages and sampling of multiple environmental predictors when conducting rigorous ecological assessments. In addition, Hill et al. [234] reported that agriculture and urbanization were consistently related with lower macroinvertebrate metric scores across the conterminous USA, presumably because of the prevalence of those human influences. Although less prevalent, metal or coal mines were reported to negatively influence fish assemblages at a threshold density as low as <0.01 mine/km^2^ [235].

The major NRSA shortcomings or gaps are: (1) not conducting the survey in the states of Alaska [236] and Hawaii or the territories of Puerto Rico, Guam, or American Samoa; (2) not surveying ephemeral or seasonally intermittent streams and rivers [237]; (3) long lag times between the field surveys and publication of the results; and (4) inadequate communication of results and implications to the USA public.

Three other national ecological monitoring programs are NAWQA (National Water Quality Assessment), NEON (National Ecological Observatory Network), and LTER (Long Term Ecological Research) sites. NAWQA biological assessments has collected ecological data from 51 study units focused on monitoring toxics and assessing the effects of urbanization and agriculture on stream and river ecosystems using fish, macroinvertebrate and algal indicators. NEON has established 27 stream and river sites that are monitored twice per summer using methods adapted from NRSA.

In addition to USEPA’s national ecological assessments, nearly all USA states have relatively active ecological assessment programs, typically focused on point sources, or egregious diffuse sources, of pollution. Five of those states have implemented particularly rigorous ecological assessment programs, some already for four decades, based mostly on macroinvertebrates but also with fish: California [238], Iowa [239,240], Maryland [241], Ohio (since 1979; [242]); and Oregon [243].

In Canada, the Canada Water Act [244] provides a mechanism for formal consultation and agreements with the provinces and territories, which has led to a monitoring focus on water quantity (via the Water Survey of Canada) and quality (water chemistry). However, there is no legislation that requires maintenance and assessment of biological condition in aquatic ecosystems at the national level. Instead, biomonitoring of freshwater ecosystems at the federal level is often used to support larger projects and developments (e.g., environmental assessments, Oil Sands development), areas of concern (e.g., Lake Winnipeg basin), national parks (e.g., for the freshwater component of the local ecological condition assessment) or trans-boundary rivers (e.g., Wolastoq | St. John River and Columbia River). Moreover, biomonitoring is used to support specific aspects of Canada’s legislation such as the Fisheries Act [245] and regulation of individual industries. Although there is no mandated systematic biomonitoring of rivers across Canada, monitoring and assessment protocols have been developed to support the various federal and regional monitoring initiatives in Canada, most notably the Canadian Aquatic Biomonitoring Network (CABIN; https://www.canada.ca/en/environment-climate-change/services/canadian-aquatic-biomonitoring-network.html).

CABIN aims to support informed decision making and cumulative effects assessment by offering a networked approach to aquatic biomonitoring using standardized methods to sample benthic macroinvertebrate assemblages. Physical habitat and water quality data are collected at the time of sampling to give a snapshot of field conditions and are supported by a suite of geospatial catchment variables. Based on a network-of-networks approach with training support, data are collected and shared by federal, provincial and territorial governments, indigenous groups, academia, industry, community groups, and non-government organizations. Although CABIN is moving towards open data by default, access to existing data requires permission from the data owner, with the exception of data collected under government agencies. CABIN data have been used to develop regional multivariate reference condition models for bioassessment, including both BEAST (Benthic Assessment of Sediment; [246]) and RIVPACS-based models [247]. Indices are often used to support the interpretation of model outputs, but CABIN data have also been used to calculate novel structural and functional diagnostic indices that expand upon the traditional assemblage-based metrics (e.g., the Canadian Ecological Flow Index; [248,249]). However, the focus for study design and site selection has generally been on smaller-extent assessments. Therefore, there has not been a targeted effort to stratify sites to answer large regional—or national-extent questions nor have there been many repeat sampling sites, which has limited temporal trend assessments.

Canadian provinces and territories have the responsibility for governance of waterways within their boundaries. That allocation of responsibility has led to different monitoring purposes and levels of legal requirements for aquatic ecosystem monitoring at the provincial and territorial level. For example, some provinces (e.g., Quebec, British Columbia and New Brunswick) have legislation or policies that mandate protection of water resources and have led to systematic monitoring of streams and rivers (e.g., [250,251]). Indeed, the New Brunswick Water Classification Regulation (currently under re-review) within the New Brunswick Clean Water Act offers a structured mechanism to potentially categorize watercourses based on both water quality and biological endpoints [252]. In contrast, other provinces and territories with more limited legislation have either adopted the national monitoring protocol (CABIN) (e.g., Newfoundland, Labrador, Northwest Territory) or developed their own monitoring protocols (e.g., Ontario—[253]; Saskatchewan—[254]]. These protocols are applied in an *ad hoc* fashion to monitor and assess activities of concern (e.g., spills, agricultural impacts and wastewater treatment) or conduct regional-extent assessments. However, to date there has not been a nation-wide effort to establish if the different biomonitoring approaches provide comparable data and assessment outcomes. Consequently, it is difficult to conduct transboundary assessments that include all Canadian provinces and territories.

Despite differences in the extent and purpose of monitoring, nearly all levels of government in Canada apply a similar suite of biomonitoring endpoints. Indeed, all provinces and territories use benthic macroinvertebrates and many also use fish, although to a more limited extent. Quebec is the only province to primarily focus on diatoms, using the Indice Diatomées de l’Est du Canada (IDEC; [255]). Ontario also has an algal biomonitoring protocol that is similar to the IDEC [256]; however, it has not been widely used to date. Most provinces and territories also lack regulatory or rehabilitation actions that are triggered by the biomonitoring and bioassessment results. Notable exceptions are the Yukon Territory, where monitoring of placer mining activities using the CABIN protocol can trigger additional monitoring and ultimately requires changes to management activities [257].

In many provinces, regional conservation agencies (e.g., watershed-based Conservation Authorities in Ontario), as opposed to provincial-level agencies, conduct the bulk of aquatic monitoring. There is also targeted monitoring data collected at more local extents, often led by community organizations, municipalities and Indigenous groups. Although these smaller-extent monitoring efforts can be limited by capacity and logistics, these initiatives are beginning to explore novel biological endpoints (e.g., the cotton strips assay; [258] for measuring standardized decomposition) that are not being used at the federal or provincial/territorial level.

In Mexico, water quality assessment of freshwaters operates federally through CONAGUA (the National Water Commission) in charge of managing water quality and water allocation in the 13 administrative hydrological regions into which the country is divided. CONAGUA implements the National Water Law (1992, amended in 2004) which states that water is federally owned, and that the federation is responsible for water allocation and for maintaining its quality and quantity.

The regional and national water quality networks included 5028 sites in 2017. More than half of the sites were deemed polluted by fecal coliforms and 33, 10 and 5% of sites were deemed polluted by COD (chemical oxygen demand), BOD_5_ (biological oxygen demand) and TSS (total suspended solids) [259]. CONAGUA has also collaborated in producing Mexican Norms (non-legally binding instruments) to calculate environmental flows in hydrological basins throughout the country and in carrying out short term freshwater macroinvertebrate-based biomonitoring in the Balsas and Bravo basins [259–261]. However, nationally, there are no laws or federally or state mandated programs for freshwater biomonitoring or restoration.

Yet, numerous biomonitoring efforts have been designed in or adapted to Mexican lotic systems since the 1970s by research institutions [262–265]. The first macroinvertebrate-based methods were based on biodiversity or saprobic indices [266,267] and were followed by binational efforts to identify biological and toxicological indicators in the Bravo River (Rio Grande) [268], in the Pescados River in eastern México [269] and the San Martín River in northern Mexico [270]. These efforts made use of the Sequential Comparative Index (SCI), the Benthic Macroinvertebrate (BMI) and Shannon–Weiner indices on macroinvertebrate data. Subsequent efforts also made use of diversity, Biological Monitoring Working Party (BMWP) and Biotic Family or Hilsenhoff indexes (IBF or IBH), including functional feeding groups as metrics for environmental evaluation [271–276]. Some approaches have integrated biotic indices with assessments of habitat condition and water quality [277,278].

Later, macroinvertebrate-based indices of biotic integrity (IBI) were developed for streams and rivers in west-central México [279,280] and central Mexico (IIBAMA, [281]), and for karst springs in the Huasteca region (eastern central Mexico) (IIBACA, [278]). These indices were modelled after Hilsenhoff’s family-level biotic index [282] and have included several parameters used elsewhere in their design (e.g., EPT). The visual environmental quality index [283], the environmental quality index [284], and the French IBGN [285] were used as benchmarks for comparison and calibration. IIBAMA has been validated [278] and applied in other basins in central Mexico [286,287]. The IBMWP was used in the Chalma-Tembembe River in central-southern Mexico [245] and the Extended Biotic Index (IBE; [288]) in the Lerma River [289]. An index developed [290] to assess the environmental impact of reservoir construction was used in the state of Hidalgo [291] and in the state of Veracruz [292].

For fish, indices of biotic integrity (IBI) were first implemented in the 1990s for west central Mexico [285]. This fish-based index included 10 metrics and was later validated for a larger region in Western and Central Mexico [293,294]. It was also modified to be applied to historical data in the Duero system [295] and to test how different sampling methods might affect MMI results [296]. IBIs based on historical fish assemblage data were also developed for the middle and lower Rio Grande/ Bravo River basin in the United States-Mexico border [297], the Nazas River (Durango, México) [298], and the Conchos River [299]. Watershed specific fish-based MMIs have also been developed for smaller tributaries of the Rio Bravo [300] and the Rio Hondo at the Mexico-Belize border [301]. Most of the above fish-based indices include a non-native species metric.

Few studies have included algae and riparian vegetation for ecological assessments in Mexican lotic systems. However algal community-based studies have been performed in tropical systems [302] and upper stretches of the Lerma River [303] and some have focused on headwaters of México City [304]. The QBR index for riparian vegetation was used in desert rivers of northern Mexico [305].

Most of the efforts described above have been initiated or implemented by individual researchers or research groups involved in short-term (typically 2–5 years), system-specific projects. They have generally not been implemented at broad extent or long-term regional efforts, but long-term biomonitoring data series exist for a few systems in the country.

All these methods require comparison of assemblages between least-disturbed sites and degraded sites. This is an important limitation in Mexico because it is increasingly difficult to find ecosystems without human impact that can function as reference sites [263]. Another limitation is the lack of information on the taxonomy and ecology of numerous endemic fish species and macroinvertebrates in several regions of Mexico.

### 2.6. Oceania

In Australia, different jurisdictions and organizations are responsible for river health monitoring and have laws that mandate the protection of aquatic ecosystems. Thus, there are many programs operating at different spatial and temporal extents using a variety of indicators [306]. For example, the Index of Stream Condition in Victoria [307] operated between 1999 and 2010 provided 5-yearly reports on Victorian streams based on hydrology, physical form, riparian vegetation, water physical and chemical quality and macroinvertebrates. In addition, the Victorian Environmental Flows Monitoring and Assessment Program (DWELP, [308]) and Wetland Monitoring Assessment Program [309] report on environmental flows (e-flows) using measurements of fish, riparian vegetation, birds and frogs, also with contribution of citizens. In south-eastern Queensland, since 2000, the Ecosystem Health Monitoring Program provides an annual report on the health of waterways using water quality, fish, macroinvertebrates, and riparian vegetation [310]. In the Australian Capital Territory, the citizen led WaterWatch Catchment Health Indicator Program (http://www.act.waterwatch.org.au/chip.html) reports annually on river condition using a combination of macroinvertebrate, physical and chemical variables and riparian condition metrics.

The Australian freshwater ecological assessments have focused mainly on rivers and streams where macroinvertebrate bioassessment is commonly used to provide one of several possible ecological indicators for the status and functioning of freshwater ecosystems. The Australian River Assessment System (AUSRIVAS) bioassessment framework [311,312] provides a standardized method for sampling macroinvertebrates and collecting environmental data and an assessment method that enabled the National River Health Program [313]. That program resulted in just one national river health survey that included 6000 sites and modelled data. That biological assessment indicated that 70% of river reaches were equivalent to reference condition and 30% were significantly impaired [314]. This survey did not include ephemeral or seasonally intermittent streams and rivers, which is an area for further research and development [315]. Catchment disturbance, elevated sediment and nutrient loads, and habitat degradation all contributed to these results. Since that initial national assessment, no updated nationally coordinated assessment of river and stream condition has been conducted, and AUSRIVAS is now largely used for targeted site assessments and state or territory-based assessment purposes [306]. Nevertheless, every 5 years the Australian Government conducts reviews the state of the Australian environment. However, this reporting on river condition remains limited because state and regional assessments are not consistent temporally and the lack of spatial coverage of sites is a major shortcoming for reporting consistent trends at this extent [316].

Over-use of water has also contributed to poor ecological condition of Australian rivers. Major water reforms in Australia over the past 20 years led to specific rules to provide water for the environment (i.e., environmental flows) and specific environmental water licenses being held by public authorities. These water holders manage large portfolios of environmental water, including within Australia’s Murray Darling Basin (MDB; 1.061 million km^2^ from Queensland, through New South Wales and the Australian Capital Territory, Victoria and South Australia), where the water is used to meet the environmental objectives of the basin-wide Environmental Watering Plan [317]. The use of the water is accompanied by major investment in ecological monitoring and evaluation funded at both State and Federal levels. Monitoring is accomplished through the Long-Term Intervention Monitoring Program [318] and the current Monitoring, Evaluation and Research Program (Flow MER program). These programs have been operating since 2014 and underpin the adaptive management of environmental water in the Murray Darling Basin and are also informing a $3.3 billion (AUD) suite of initiatives to support implementation of the Murray–Darling Basin Plan, most of which focus on improving river health [319].

Annual monitoring of fish assemblages, groundcover vegetation diversity and stream metabolic responses to watering actions is undertaken using standardized methods within seven catchments across the Murray Darling Basin and is supplemented with less frequent catchment specific monitoring of waterbirds, frogs and tree condition [320,321]. The basin-extent monitoring program to evaluate the effectiveness of the Murray-Darling Basin Plan is tracking progress towards long-term basin targets associated with environmental watering plans and expected environmental outcomes of the basin-wide environmental watering strategy. The targets and outcomes fall under four broad themes: river flows and connectivity; native vegetation; waterbirds; and native fish. Tree stand condition monitoring since 2013 is providing annual snapshots of the condition and extent of native riparian and floodplain vegetation across the basin [318]. The Murray-Darling Basin Fish Survey (built on past programs such as the Sustainable Rivers Audit; [322]) is monitoring changes in the condition of native fish based on presence, movement and population structure (MDBA 2017). The South-East Australian Aerial Waterbirds Survey, which has been running for 37 years, measures the distribution and abundance of waterbirds annually. Some research and development have been undertaken in Australia regarding the use of macroinvertebrate traits [323] and a traits database (https://ausrivas.ewater.org.au/index.php/traits), and DNA biomonitoring tools (e.g., macroinvertebrates—[324]; invasive and threatened species—[325]) but the methods have not yet been adopted widely for routine biomonitoring.

The Commonwealth Environmental Water Office has provided ~$10 million for over 50 short-term intervention monitoring (STIM) projects since 2011. These projects typically run for several months to four years. Each project is designed to help address a priority ecological knowledge gap at a local or catchment extent to inform adaptive environmental water management. Each project falls under one of the four themes listed above. Information collected from short-term intervention monitoring has helped to identify where policy and operational constraints have impeded progress towards basin-extent environmental targets and outcomes. Some of these projects have helped to demonstrate where in the basin environmental water was not adequately protected from extraction. Policy and legislative changes to improve how water is provided for the environment in the northern Murray-Darling Basin have been informed by STIM project findings.

In New Zealand (NZ) regular ecological assessments of freshwaters is required by some laws, primarily the Resource Management Act (1991). There is no national monitoring program, although the National Institute of Water and Atmospheric Research (NIWA) annually monitors the lower reaches of 77 primarily larger rivers throughout the country. As in Australia, freshwater ecological assessments are generally devolved to the regional government. Each of the 16 Regional Councils (or equivalents) are required to conduct regular, usually annual, State of Environment monitoring. The frequency of monitoring, number of sites and parameters measured vary between councils depending on political, economic, social and cultural pressures. Furthermore, some city councils of larger cities (e.g., >50,000) have limited annual monitoring programs. Some other agencies (e.g., Department of Conservation, Fish and Game NZ) have targeted, often species specific, monitoring. Several rehabilitation projects funded by private organizations or individuals may conduct short-term, limited parameter monitoring.

Freshwater ecological assessments in New Zealand have traditionally focused on rivers and streams. Historically water quality parameters were measured (e.g., pH, dissolved oxygen and nutrients, temperature, turbidity, and human health bacteria) but macroinvertebrates are now the most commonly monitored biological assemblage [326]. Algae, macrophyte biomass and cover, fish, and functional indicators (e.g., GPP, decomposition rates) are less frequently monitored. About 50% of freshwater fish are introduced and many are nuisance or pest species that are only monitored in some regions.

Typically, a range of diversity and biotic indices are used for reporting benthic invertebrate data. Most councils collect semi-quantitative, relative abundance, or fixed count data. These indices include total taxonomic richness, EPT (relative abundance or %), and a New Zealand tolerance index the MCI (Macroinvertebrate Community Index; [327]).

Most regional councils aim to include reference conditions and reference sites; however, some struggle to find sufficient reference sites particularly in lowland, intensively farmed regions where unimpacted, or best condition sites are rare or absent. The number and parameters measured in monitoring programs are often limited by resources, both funding and expertise. Although there are several recommended standard protocols for benthic invertebrates [328] and other geomorphological parameters [329,330] numerous councils continue to use their own variations and no national consistency exists, although a program of National Environmental Monitoring Standards (NEMS) is being developed. Invasive species are rarely monitored, although the NZ Fish and Game Councils oversee the salmonid fishery and conduct semi-regular surveys of redds and some fish counts (primarily brown and rainbow trout and sockeye salmon), however, this is for recreational fishing purposes, not stream health.

Regional councils are required to make the results of State of the Environment reports available to the public. Nationally synthesized interpretations are available via the Land and Water Aotearoa website (https://www.lawa.org.nz/). This site has data for over 1450 sites throughout the country and results can be viewed for an assessment of current status and trends.

Considerable controversy exists over the condition of New Zealand rivers. Increased dairy farm intensification has led to significant increases in nutrient concentrations, toxic algae, and human health bacteria. In 2017, 61% of 175 monitored rivers showed worsening nitrate concentrations. Approximately 70% of fish species, 33% of benthic invertebrates, and 33% of aquatic plants are threatened or endangered.

## 3. River Rehabilitation

### 3.1. Asia

In Japan, river rehabilitation was boosted by the Nature-oriented River Works (NRW) in 1990 and the amendment of the River Law in 1997, when Conservation and Improvement of the River Environment was inserted as the principal goal [80]. The results of NCRE have been used to create the Information Map of River Environment, which is based on a vegetation map with habitats and rare species listed. The maps reveal trends in Japanese rivers and were used to a great extent in river rehabilitation projects [331].

Although the NRW has been conducted in more than 23,000 cases until 2004 [80], this approach was basically only nature-friendly river engineering. Then, the Act on the Promotion of Nature Restoration in 2002 promoted river rehabilitation in Japan, for instance, the Kushiro [332] and Kamisaigo Rivers [333] where the river was widened, and the number of species increased. In addition, by involving the public from the planning stage, a high level of maintenance and management with low cost have been achieved. More examples are shown in Table S2.

Recently, river rehabilitation is spreading from the river to the basin to the ecological network [334] and the implementation of green infrastructure. Japan’s river rehabilitation efforts are also dedicated to recovery from disasters. An environmentally friendly disaster recovery manual has been prepared, supported by designated river rehabilitation advisors.

In the Republic of Korea, except for very small streams, river management is led by the Regional River Management Agency of the Ministry of Land, Infrastructure and Transport in accordance with the River Act. Under the 2018 Water Environment Conservation Act, the government has developed and operated national river ecological rehabilitation programs. In addition, the Ministry of Environment prepared an administrative and financial support system through the Guidelines for the Promotion of Ecological River Restoration Projects. Those guidelines aimed to enhance the ecological value of rivers in accordance with the government’s fiscal decentralization in 2020.

Korea achieved great results in terms of flood control, water supply and water quality management by the mid-1990s. However, river ecology did not receive much attention until the late 1990s. The Yangjaecheon River in Seoul, was the first urban river to be rehabilitated in Korea by private capital inducement, and the project received favorable reviews from citizens. Since then, river rehabilitation has been gradually expanded nationwide with state support, including the Anyangcheon River in Anyang and the Cheonggyecheon River in Seoul. Increasingly, river naturalization focused on aquatic ecology has become a major concern in river management. One interesting example is the day-lighting of the Cheongyecheon River, which used to be covered by a highway and is now a river-side park (https://www.landscapeperformance.org/case-study-briefs/cheonggyecheon-stream-restoration) (Table S2).

Singaporean river rehabilitation has primarily been driven by the government. The most significant project has been the naturalization of 3.2 km of the Kallang River (Bishan-Ang Mo Kio Park Project) which was a concrete canal (https://ramboll.com/projects/singapore/bishan-park) (Table S2). Monitoring has recorded 59 bird species, 22 dragonfly species, and a family of five freshwater otters using the rehabilitated reach.

In China, the need to rehabilitate water quality to national standards was driven by several statutes. The 1984 Law on the Prevention and Control of Water Pollution (amended in 1996 and 2008) introduced the need to sustain the ecological function of water bodies in developing, using, regulating, and allocating water resources. The 1988 Water Law (revised in 2010) included: (1) a reward system for the institutions and individuals that make outstanding achievements; (2) the need to consider the protection of the ecological environment when developing hydropower stations, and (3) the need to build fish passage facilities.

However, these laws are only general provisions and do not include technical requirements. That leads to difficulties in implementation and differences in the quality of the rehabilitation projects. A bibliographic search in >880 references on river ecological rehabilitation in China from 2000 to 2020 showed that only 8% of the projects use biological habitat, diversity or integrity as targets. In addition, many projects simply improve water quality, aesthetics, and consider only short-term goals.

Thus, in 2015, the Ministry of Water Resources issued the Guidelines for Aquatic Ecological Protection and Restoration Planning with technical requirements for the rehabilitation of aquatic ecosystems. Yet, the impact of these guidelines on the quality of projects is still poorly assessed, as these standards do not mention how baseline and target objectives are determined, and the final decision is made on case-by-case judgments.

In addition, China put forward in 2017 national strategies for the ecological protection of the Yangtze River and in 2019 for the Yellow River basin, China’s second-largest river. The Yangtze River Protection Law is the first legislation of the era of integrated river basin management in China. The protection of the Yangtze River involves the rational allocation, development, and use of water resources. From the perspective of ecological protection, it pays attention to water pollution prevention and control, water quality improvement, water ecological protection, water risk prevention, and water security.

Currently, there are no extensive river ecological rehabilitation projects (but see Table S2). Yet, the Plan for The Protection and Restoration of Major National Ecosystems (2021–2035) published in 2020, clarifies the overall requirements and major targets for ecological protection and rehabilitation across the country by 2035. It puts forward key tasks, policies, and measures for major projects, and forms a basic framework for promoting the protection and rehabilitation of major ecosystems in the country.

### 3.2. Africa

In South Africa, rehabilitation efforts can be triggered if the RQOs established by the National Water Act are not being met. Yet, implementation has been challenging for several reasons, including the limited capacity for enforcement and compliance monitoring and appropriate sanctions on polluters, and a lack of coordinated rehabilitation efforts. The best example of a successful rehabilitation program in South Africa is the Working for Water program, which aimed at removing invasive non-native vegetation from catchments across the country. Buch and Dixon [335] and Marais and Wanenburgh [336] have shown the net benefit of the program. In the rest of Africa, river rehabilitation is almost non-existent.

### 3.3. Central and South America

South American countries have no legislation requiring the implementation of rehabilitation programs or projects. However, existing legal statutes establish responsibilities and mandate mitigation and rehabilitation actions after environmental accidents like oil spills or dam failures. Programs to rehabilitate watercourses are usually initiated by state and local governments using federal resources or international agencies but there are also examples of private initiatives [337]. Often the most successful examples come from community initiatives rather than government initiatives [338].

The main actions in South America address the implementation of sewage collection systems, rehabilitation of riverbanks through engineering and bioengineering techniques, rehabilitation of riparian vegetation, and flood mitigation [337–341]. In Argentina and Brazil, some studies have also created analytical bases to support future rehabilitation (e.g., [342–346]).

Examples of successful initiatives at the local level include the rehabilitation of urban streams in Cuenca, Ecuador [347], and Belo Horizonte, Brazil [348,349]. The followup monitoring was implemented through the Recurb Project of the Federal University of Minas Gerais, which assessed the effect of the rehabilitation of three streams over 10 years [119,350]. Results highlighted the improvement of water quality and species richness, composition and assemblage structure of benthic macroinvertebrate assemblages, and appearance of new sensitive taxa. In addition, there was a wide acceptance of interventions by the urban population assessed through interviews.

Important contributions to the protection and recovery of river basins have been related to the improvement of water for human consumption. This is the aim of the Latin American alliance which includes Central and South America and Caribbean countries [351]. A successful example is the Quito Water Fund (FONAG) which helped to recover the ecological quality of Paramo streams that are the source of potable water for the city [352]. Another example is the technical cooperation that Peru maintains with South Korea for rehabilitating rivers (https://www.ana.gob.pe/normatividad/resoluciones-ana/normas-importantes-instrumentos-gestio).

However, in general, initiatives in South America have achieved more societal than ecological values [338]. One of the main reasons may be the absence of ecological standards for this type of intervention because most goals are linked to aesthetic and water quality aspects, sediment control and flood events, or cleaning up oil and mine tailings spills [337,338,340].

### 3.4. Europe

According to the Water Framework Directive, the RBMPs, revised every 6 years, must include the Programs of Measures for all the water bodies falling below Good ecological quality status. All member states reported basic measures to reduce diffuse pollution. Although many measures and volunteer actions from farmers focused on improving water quality, this has not been sufficient. Nonetheless, ammonium and BOD_5_ decreased in European rivers from 1992 to 2018 (78% and 54% respectively) mainly as a result of improved waste-water treatment (https://www.eea.europa.eu/data-and-maps/data/waterbase-water-quality-icm).

Reduced flows resulting from water abstraction and water retention by dams (with consequences for longitudinal connectivity) have become greater concerns and are worsened by climate warming, especially in southern Europe [353–355]. Therefore, the European Commission recommends elaborating Drought Management Plans. Water abstraction has been addressed by implementing controls and reviewing licenses which have improved conditions in some countries between the 1st and 2nd RBMP. Regarding hydromorphological pressures, the improvements have been minimal although many countries plan to install fish passages or remove barriers.

In addition, other rehabilitation projects have been completed that were not included in the RBMP. The European Centre for Rivers Restoration (ECRR) is a 20 years old international network that synthesizes knowledge and supports the development of best practices for successful river rehabilitation and management. It focuses on ecological, physical, spatial, and management measures and practices. It also provides a Manual of River Restoration Techniques (https://www.therrc.co.uk/manual-river-restoration-techniques), developed by the UK Rivers Restoration Centre.

The European projects, RESTORE, REFORM and FORECAST, contributed to the RiverWiki database (https://restorerivers.eu/wiki/), which is being continuously updated and currently includes 1325 river rehabilitation projects in 31 countries. Most cases reported are from northern Europe, especially from the UK (>700), but there are also many projects taking place in the Netherlands, France, Spain, Austria, Italy, and Denmark. Most measures applied are bank stabilization and reshaping, creation of fish passes, dam removals, secondary channels creation, and riparian vegetation plantings. Many projects address several problems simultaneously reflecting the co-occurrence of multiple stressors in most European rivers [355–357].

In some case studies, response variables are monitored such as macroinvertebrate, fish and macrophytes assemblages, hydromorphology and water chemistry, either before and/or after the rehabilitation actions. Yet, from the database of 1325 study cases, only 161 indicated that ecological monitoring was performed. Indeed, after two decades of WFD history in Europe, the procedure of rehabilitating rivers in Europe remains poorly standardized, with the effects of the measures on ecosystems and biodiversity being monitored in only a fraction of cases [358]. Although bioassessment increases the total cost of any rehabilitation project, it is still remarkable that the values of collecting such data are easily dismissed, particularly considering the high costs of rehabilitating rivers [359].

From the relatively few rehabilitation projects that have been monitored before and after the measures were implemented, valuable lessons have been learned. Whereas improvements in hydromorphology can be achieved with small measures in the short term, to improve biological assemblages significantly, large river sections must be recovered and catchment-wide measures and long recovery periods should be expected [360,361]. Furthermore, success is highly dependent on the design of the on-site measures and the interplay with other measures elsewhere in the catchment [362]. One successful example is the rehabilitation of the longitudinal continuity of the Mondego River (Portugal), which was achieved with the construction of five nature-like fish passes and one technical fish pass (vertical slot fish pass at Coimbra Dam). The barrier treatments opened migratory routes and 45 km of habitat for diadromous fishes (*Petromyzon marinus*, *Alosa alosa*, among others; [363]). About 1.5 million fishes are annually recorded using the fishways and a 90-fold increase of sea lamprey larval abundance has resulted from these actions [364]. The rehabilitation was also complemented by the management of related commercial fisheries, including the implementation of an intermediate fishing closure for sea lamprey and shads during the peak of the spawning season [365]. Other examples of relevant rehabilitation projects in Europe are indicated in Table S2.

Up to now, several independent and national initiatives have been initiated, including an integrated project planning framework [366], diverse guidelines and strategies (e.g., Spanish National Strategy for River Restoration—[355], and the RiverWiki database). However, these frequently cover only a fraction of the existing projects or are aimed at particular countries or regions. To further promote nature rehabilitation, the European Commission published in May 2020 the Biodiversity Strategy for 2030: Bringing Nature Back into our Lives, which is focused on nature rehabilitation. Regarding rivers, the strategy considers that natural functions must be recovered to achieve the Good ecological condition expected under the WFD. The European Commission aims to rehabilitate 25,000 km of rivers by, among others, removing structures such as obsolete dams.

### 3.5. North America

To date, the results of the NRSA national and ecoregional assessments in the USA have not led directly to any major stream or river rehabilitation program. However, the 1972 Clean Water Act authorized funds and technical expertise that led to substantial improvements in lotic ecosystem quality across the entire USA, particularly as a result of industrial and municipal waste treatment. Unfortunately, there was no national rigorous ecological monitoring program for assessing the biological results of those efforts. Nonetheless, states with long-term ecological monitoring programs offer pertinent results. For example, Ohio EPA [367] determined that the percent of river kilometers assessed statewide that met aquatic life standards increased from 20% in 1980 to 75% in 2018. Following basin-wide waste treatment in Oregon’s Willamette River, the numbers of fish species and intolerant fish species increased, whereas the number of tolerant fish species decreased in the mainstem river [368].

Most recent rehabilitation projects have been localized, and very few of those have been accompanied by rigorous ecological BACI (before after control impact) monitoring, despite billions of dollars spent on them [369]. Kauffman et al. [370] suggested rehabilitating western USA rangelands and riparian zones simply by excluding livestock and allowing time for the catchments, streams and riparian zones to recover naturally. Palmer et al. [371] recommended that ecological success of rehabilitation projects should be based on (1) what constitutes an ecologically healthy river, (2) improved ecological condition, (3) minimal continued maintenance, (4) minimal harm during the rehabilitation project, and (5) BACI ecological assessments and public reporting. Although Roni et al. [372] examined 345 stream rehabilitation studies, firm conclusions about the effectiveness of most of them were hindered because of insufficient ecological and historical information, failures to understand catchment processes, and poor monitoring designs. However, reconnecting isolated habitats, rehabilitating floodplains, and improving instream habitat often increased local fish abundance. They recommended protecting high-quality streams and ensuring stream connectivity and watershed processes before attempting instream rehabilitation projects. Similarly, Stranko et al. [373] reported that fish and benthic macroinvertebrate diversity of rehabilitated and unrehabilitated urban stream sites were similar and less than in reference stream sites because of overall catchment conditions. Bernhardt and Palmer [374] concluded that reach-extent mitigations are ineffective for reversing the chemical, physical and hydrological alterations that limit sensitive taxa and water quality improvement. Nonetheless, there are examples of successes, mostly focused on fish assemblages through the addition of instream structures (e.g., large wood and rock; [375]) channel reconstruction, naturalized instream flows, fish passage improvements, and restricted livestock grazing [376–378].

Unregulated agriculture and insufficiently regulated urban effluents are the current major threats to attaining good ecological condition in most USA lotic ecosystems [229,234]. Additional threats stem from metal and coal mining [235], unregulated oil and gas production [379], unregulated livestock grazing [380], dams and diversions [381], exclusion of headwater streams and wetlands from CWA protections [382], and climate change [383]. However, the drivers of these pressures are continued economic and population growth [384], significant declines in state agency staffing and funding [385], and direct and indirect weakening of state and federal environmental standards [386,387].

In Canada, river rehabilitation is limited in scope and generally does not arise as a consequence of systematic monitoring. However, river rehabilitation activities do occur at the national level as a consequence of violations of the Canadian Fisheries Act [245]. Moreover, water-related legislation of some provinces (e.g., British Columbia, New Brunswick, and Quebec) stipulate that should a river’s water quality or biology be impaired then rehabilitation efforts should be undertaken. For example, in Quebec, if a river is considered impaired, then legislation is in place that could lead to the initiation of specific rehabilitation efforts of that river (Act to Affirm the Collective Nature of Water Resources and to Promote Better Governance of Water and Associated Environments 2009). Most often, river rehabilitation is initiated at the municipal or regional level as a consequence of public interest-driven by community groups or non-government agencies in response to perceived urban development or agricultural impacts. Consequently, funding and objectives of rehabilitation efforts vary widely in scope and frequency at the national and provincial/territorial levels. In general, rehabilitation efforts tend to emphasize channel design and fish habitat aspects of rehabilitation as well as riparian vegetation. Other common foci of river rehabilitation projects across Canada include the removal of small dams and barriers, restriction of livestock access to streams, and upland best management practices aimed at reducing diffuse-source loadings of pollutants to streams. Although many of these projects are considered successful, the lack of specific and consistent legislation at the national and provincial/territorial levels has led to the piecemeal implementation of river rehabilitation and project success is often hampered by larger-extent issues (e.g., water quality) that prevent the full benefits of local rehabilitation projects from being realized from a river health perspective.

Mexico is among the top 5 countries with the highest natural forest loss in the last 20 years [388]. Similarly, Mexican lotic systems continue to suffer severe alterations, and numerous reaches are incapable of sustaining a native biota given a variety of stressors, including severe ecohydrological alterations [389]. This calls for urgent protection and rehabilitation measures. Contrary to other North American nations, Mexico lacks a national program or policy for ecological rehabilitation [390], despite agreeing to national and international treaties and projects promoting habitat rehabilitation (e.g., Aichi Biodiversity Targets, Climate Summit 2014) [388]. Rehabilitation efforts remain isolated, costly, and with low effectiveness [391]. Freshwater rehabilitation efforts have been implemented by independent research groups working collaboratively with NGOs and government agencies in selected ecosystems, such as the riparian areas of the Lacandon rainforest [392]. Because 80% of Mexico’s land is privately owned [393], rehabilitation efforts in Mexican freshwater ecosystems are driven by socioeconomic limitations [388]. However, the implementation of Environmental Water Reserves (EWRs) is an increasingly possible rehabilitation strategy, based on the Mexican Environmental Flows Norm. An EWR is an annual volume of water that is allocated to remain in the environment for up to 50 years. Currently, there are 295 basins with EWRs, and the national water program 2020–2024 established a goal of 448. Macroinvertebrate, riparian vegetation, and fish protocols for environmental flow assessments have been developed as part of the Building Block Methodology [394].

### 3.6. Oceania

Numerous rivers in southern and eastern Australia and New Zealand were severely affected by human activities before bioassessment programs were underway. Examples are: extensive sands deposited in lowland river reaches throughout south-eastern Australia from the late 1800s [395], flow alteration from water extractions and dam-building starting in the mid-20th century [396], and the spread of European carp [397] and other pest fish.

Rehabilitation policies and actions in Australia are often driven by either a response to the conservation status of local biota (e.g., the endangered Macquarie Perch, Table S2) [398] or to legislation, regulations, and plans aimed at limiting or reversing environmental degradation caused by excessive nutrients, sediments, salinity, organic carbon and cyanobacteria or to restore flows. Many jurisdictions have environmental flow objectives that arise from the Water Resources Act 2007 [399], which are also a policy driver for rehabilitation.

The factors driving river rehabilitation can be numerous. For example, the Murray Darling Basin Plan was built on the National River Health Initiative and other programs such as the Sustainable Rivers Audit (SRA) [322] and the Land and Water Resources Audit (LWRA) [313]. The culmination of increasingly evident water issues (emphasized by drought) and associated bioassessment programs indicated the basin was in poor ecological condition.

In New Zealand, there is no national legislation requiring river rehabilitation, although the National Policy Statement on Freshwater Management requires Councils to maintain or improve water quality in all waterways. There are limited examples of rehabilitation of waterways being undertaken by the Government (e.g., Project River Recovery, Tui Mine Remediation Project), some by Government-Industry partnerships (e.g., Living Water—a Department of Conservation and FONTERRA partnership in four catchments). However, most projects are undertaken by community organizations or individuals. Ecological monitoring is sporadic, often short-term, and measures aiming at ecological recovery are rare. Often the aims are either not clearly defined or may consist of conflicting or unrealistic goals. Riparian planting dominates rehabilitation projects, increasingly fish passage barriers are being targeted, but dam removal has not yet started in NZ.

## 4. Conclusions

Existing ecological monitoring in the world is driven by different reasons: (1) the need to determine river condition; (2) environmental impact assessment projects; (3) assessing the effects of rehabilitation projects, and (4) citizen-science projects aimed at increasing awareness and educating. Independently of the reasons, ecological monitoring is widely and consistently implemented in Europe, North America, and Oceania. It is not widely implemented in South America, Africa, or Asia—despite occurring in some countries (e.g., Republic of Korea, Japan, South Africa). Thus, it is not currently possible to have an accurate overview of the ecological condition of rivers worldwide. From what was reported, rivers in all continents are impaired by excessive nutrient concentrations, migration barriers, altered flow, sedimentation, and alterations or removal of riparian forests. The biological condition of rivers is less than Good for more than half of the water bodies in Europe and the USA, and in Japan and New Zealand, a striking loss of biodiversity was observed in recent decades. However, to have a realistic overview of biodiversity and river condition and response to rehabilitation measures, long-term and large-extent regular monitoring programs are needed.

Striking differences in ecological monitoring approaches among nations were detected especially in Africa, Asia, and South America, where national/regional bioassessment frameworks are not yet widely developed and implemented. Monitoring implementation is limited by (1) coordination across institutions and between stakeholders; (2) scientific knowledge gaps, and (3) insufficient resources. Countries, where rigorous ecological monitoring is not occurring, could benefit from the European Union’s experience with the implementation of the WFD and USA’s NRSA and be encouraged to develop biological monitoring. These measures would allow for a more complete overview of the ecological condition of rivers worldwide. This is particularly relevant for large transboundary rivers (e.g., Colorado, Grande, Yukon, Columbia, St. Lawrence, Rhine, Danube, Nile, Congo, Mekong, Amur). Yet, to be successful such international actions require better integration of nations ecologically, economically and politically, because some rivers drain across countries with very different conditions. Examples are the Amazon (draining parts of Bolivia, Peru, Ecuador, Colombia, and Brazil), Parana (draining parts of Argentina, Brazil, Paraguay, and Uruguay), and Africa’s Niger, Nile, Congo, and Zambezi Rivers draining portions of 17 nations. Asia’s Shatt al Arab, Indus, Brahmaputra, Mekong, and Amur rivers connect 13 countries.

Benthic macroinvertebrates are the most commonly used assemblage in bioassessment and quality status is determined mostly through MMIs and predictive models. However, other assemblages (i.e., fish, macrophyte, and microalgae) are also regularly used in Europe, the USA, Australia, New Zealand, and Japan (e.g., [17,58,80,83,160,400]). Other assemblages have had a restricted use such as waterbirds in the Murray-Darlin basin, Australia, or in the USA [401]. Nonetheless, given strong budget limitations, it is usually necessary to restrict the number of assemblages used. In most cases, macroinvertebrate and fish assemblages respond strongly to multiple types of disturbances (or rehabilitation), thus they should be used for large-extent and long-term programs. This would require defining appropriate river typologies, predictive models, and adapting or developing new indices based on the new reference conditions where they do not exist (for example for intermittent and saline rivers).

A major shortcoming in existing bioassessments, especially those based on macroinvertebrates, is not considering the presence of non-native or invasive species or as a negative effect, and often they are simply included as one more taxon. On the other hand, some fish indices already negatively weight those taxa. Because non-native species are currently a major stressor in river ecosystems worldwide, resulting in the destruction or reduction of local biodiversity and altering ecosystem services, we recommend that they be consistently monitored and accounted for when determining river biological quality.

Finally, there are problems with the spatial extent and design of most ecological monitoring programs. National or continental statutes, such as the European Water Framework and the USA Clean Water Act focus on all waters. Typically, most ecological monitoring in other nations and continents is performed at a local or basin extent and guided by local or regional statutes or specific pollution concerns. It is unlikely that combining information from multiple local programs with differing survey designs, sampling methods and indicators can serve to rigorously assess river health at national—let alone continental—spatial extents [402]. Therefore, to obtain a broader overview of river quality, extensive monitoring networks and standardized sampling and reporting systems are important. On the other hand, if the aim is rehabilitating an impaired river or stream reach, a finer resolution monitoring program and appropriate indicators for the problem are advisable. Such reporting is particularly important to enable ecological assessment of changes in land and water use, and climate change given that they have broad-scale and multi-jurisdictional impacts. Above all, any ecological assessment program must be tied to the ecological objectives and resource management framework.

Considering the difficulty of implementing extensive regular biological monitoring and the poor knowledge of biodiversity in many countries, molecular-based assessments from bulk (invertebrates, diatoms) or environmental (water) samples may offer a promising alternative at lower costs in the near future [403,404].

Regarding rehabilitating impaired rivers, many successful efforts have occurred in Europe, North America, Australia, and some Asian countries (e.g., Japan, Korea, Singapore). The largest percentage of rehabilitation works have aimed to recover water quality, facilitate fish migration, or restore flows or riparian vegetation. In fewer cases, rehabilitation has targeted channel naturalization or wetland ecosystem functioning and flooding cycles.

In general, river rehabilitation has been hampered by many factors (e.g., [355]; Table 1). There is little specific legislation focused on river rehabilitation and there are substantial economic and political constraints for implementing projects. Private land ownership and ineffective government regulation and enforcement hinder projects. While minimal awareness and technical knowledge among water managers and decision-makers may hinder the implementation and effectiveness of rehabilitation projects, in some countries social/community constraints are often barriers to achieving the desired ecological outcomes. Pre- and post-rehabilitation ecological monitoring data are rarely available, meaning that there is insufficient ecological and historical information regarding the effectiveness of various types of rehabilitation projects. Typically, projects are focused on local stream sites or reaches; however, those areas are also often limited by upstream water quality and flow regime limitations. Well-established targets are missing or inappropriate regarding both structural and functional ecological indicators. More rehabilitation should be targeted on reducing non-native invasive species, which are extremely difficult to remove once they become widely established. Standard methods detailing the technical requirements for successful projects are lacking. Increased public education regarding the importance of river ecosystems and their ecosystem services is needed for motivating rehabilitation projects [405]. The public should be at the forefront of this effort because governments are often responsive to public pressure when formulating their rehabilitation agendas. Finally, much clearer connections between routine biological monitoring and rehabilitation are necessary.

Our study highlighted the need for international teams, without which it seems difficult to understand the key dimensions of rigorous biological monitoring and rehabilitation globally, because of the publication of results and strategies only locally available and in native languages. International collaboration is thus essential to promote river biological monitoring and rehabilitation globally. Therefore, we recommend establishing a set of international working groups to draft legal statutes, rigorous biological monitoring field and laboratory protocols, citizen-scientist monitoring protocols, numerical biological indicators, and cost-effective river rehabilitation protocols. We also recommend making such expert teams available through the United Nations Environment Program to aid the extension of biomonitoring, bioassessment, and river rehabilitation knowledge globally. The teams should include social scientists that are sensitive to the different cultural perspectives and values that hinder biomonitoring implementation.

## Supplementary Material

Supplement1 Table S1: Examples of ecological monitoring networks/programs of rivers and streams implemented in the World (based on the countries considered by this study) by official authorities or “seed programs” by research teams (where no official program is available); Table S2. Examples of rehabilitation of rivers around the world aiming the improvement of the biological assemblages.

## Figures and Tables

**Table 1.** Factors that enable successful ecological river restoration programs.

Enablers	Description
Strong mandate	This may be either political or public. Must serve a purpose or defined ecological and social need, that fits within a management and policy framework
Political context	Requires high priority (national, state, or regional) policy drivers and responsibility at that policy level.
Governance and funding	A program that has a mandate needs dedicated program funding, coordination, and the associated governance structure to support it. This is particularly important for programs that by their nature may require a long-term commitment.
Clear ecological objectives	“SMART” (specific, measurable, achievable, relevant, and timed) ecological objectives are required [406]. Programs need appropriately scaled ecological objectives designed to deliver management outcomes that are relevant to the environment, policy, and investment. Consider interim measures of success where rehabilitation may take a long time (decades) to be realized.
Fit for purpose	The program must be tied to the ecological objectives and resource management framework. What will be realistically achieved within what timeframe?
Trust and communication	Build trust and good communication among stakeholders. This is important to provide the social license to manage adaptively (because the program may evolve over time) and to build knowledge pathways.
Social license	A program that has public support will be easier to enable. Enlist social science expertise.
Ecological knowledge	Success will depend on a good foundation of ecological knowledge. Partner with those having ecological knowledge e.g., industry, universities, and research organizations.
Technical knowledge	Use current and relevant methods based on scientific evidence to increase the likelihood of successful ecological outcomes.
Measures of success	An associated ecological assessment program that is linked to the objectives and appropriate ecological timeframes to assess the effectiveness of the rehabilitation efforts. Need to identify the key biological indicators for ecological assessment and rehabilitation.

## Data Availability

No new data were created or analyzed in this study. Data sharing is not applicable to this article.

## Abbreviations

ASPT: Average Score per Taxon
AUSRIVAS: Australian River Assessment System
BACI: Before After Control Impact (monitoring design)
BBI: Belgium Biotic Index
BCG: Biological Condition Gradient (USA)
BDO5: Biological Dissolved Oxygen
BEAST: Benthic Assessment of Sediment
BMI: Benthic Macroinvertebrate Index
BMWP: Biological Monitoring Working Party
CABIN: Canadian Aquatic Biomonitoring Network
CEE: European Economic Community Index
COD: Chemical Oxygen Demand
CONAGUA: Comisión Nacional del Agua (Mexico)
CONAMA: Conselho Nacional do Meio Ambiente (Brasil)
CWA: Clean Water Act
DARLEQ2: Diatoms for Assessing River and Lake Ecological Quality (UK)
DNA: deoxyribonucleic acid
DWELP: Victorian Environmental Flows Monitoring and Assessment Program
ECRR: European Centre for Rivers Restoration
eDNA: environmental DNA
EFI+: European Fish Index
EPT: Ephemeroptera, Plecoptera, and Trichoptera
EQS: Ecological Quality Status
EU: European Union
EWRs: Environmental Water Reserves (Mexico)
F-IBIP: Indice Piscícola de Integridade Biótica para Rios Vadeáveis de Portugal Continental
FONAG: Fundo para a Protecção da Água (Quito Water Fund)
GDI: Generic Diatom índex
HKHbios: Hindy Kush-Himalayan biotic score
IBD: Indice Biologique Diatomées
IBGN: Indice Biologique Global Normalisé
IBI: Indices of Biological Integrity
IBMR: Indice Biologique Macrophytes Rivières
IBMWP: Iberian BMWP
IC: Intercalibration Exercise
IDEC: Indice Diatomées de l’Est du Canada
IMMi: Iberian Mediterranean Multimetric Index
IPS: Indice de Polluosensibilité Spécifique
IPtI: Índice Português de Invertebrados
LEAFPACS2: River Assessment Method Macrophytes (UK)
LTER: Long Term Ecological Research
LWRA: Land and Water Resources Audit (Australia)
MDB: Murray Darling Basin (Australia)
MLIT: Ministry of Land, Infrastructure, Transport, and Tourism (Japan)
MMI: Multimetric index
MMIF: Multimetric Macroinvertebrate Index Flanders
MMIZB: Multimetric Index for Zio River Basin
MTR: Mean Trophic Rank
NASS: Namibian Scoring System
NAWQA: National Water-Quality Assessment (USA)
NCRE: National Census on the River Environment (Japan)
NEMS: National Environmental Monitoring Standards (New Zealand)
NEON: National Ecological Observatory Network (USA)
NIWA: National Institute of Water and Atmospheric Research (New Zealand)
NRSA: National Rivers and Streams Assessment (USA)
NRW: Nature-oriented River Works (Japan)
OKAS: Okavango Assessment System
ORASECOM: Orange-Senqu River Commission
QBR: Index of Riparian Quality
RBMP: River Basin Management Plans
REMP: River Ecostatus Monitoring Programme
RICT: River Invertebrates Classification Tool (UK)
RIVPACS: River Invertebrate Prediction and Classification System
RQOs: Resource Quality Objectives
RVI: Riparian Vegetation Index
SAFRASS: Southern African River Assessment Scheme
SASS: South African Scoring System
SCI: Sequential Comparative Index (invertebrates)
SIGNAL: Stream Invertebrate Grade Number-Average Level index
SRA: Sustainable Rivers Audit (Australia)
STAR_ICMi: Intercalibration Comon Metric index
TDI: Trophic Diatom Indices
TSS: Total Suspended Solids
USEPA: United States Environmental Protection Agency
WFD: Water Framework Directive
WSA: wadeable stream assessment (USA)
ZISS: Zambian Scoring System
