# Supplementary material for: The Biological Assessment and Rehabilitation of the World’s Rivers: An Overview
Source: Water (Basel). Author manuscript; Available in PMC 2022 Jan 31. (PMC8048141; doi:10.3390/w13030371)
Supplement: Supplement1 — Table S1: Examples of ecological monitoring networks/programs of rivers and streams implemented in the World (based on the countries considered by this study) by official authorities or “seed programs” by research teams (where no official program is available); Table S2. Examples of rehabilitation of rivers around the world aiming the improvement of the biological assemblages. [file NIHMS1672837-supplement-Supplement1.pdf]

water

MDPI

Review

The Biological Assessment and Rehabilitation of the World's Rivers: An Overview

Maria João Feio [1,*], Robert M. Hughes [2,3], Marcos Callisto [4], Susan J. Nichols [5], Oghenekaro N. Odume [6], Bernardo R. Quintella [7,8], Mathias Kuemmerlen [9], Francisca C. Aguiar [10], Salomé F.P. Almeida [11], Perla Alonso-EguíaLis [12], Francis O. Arimoro [13], Fiona J. Dyer [5], Jon S. Harding [14], Sukhwan Jang [15], Phillip R. Kaufmann [3,16], Samhee Lee [17], Jianhua Li [18], Diego R. Macedo [19], Ana Mendes [20], Norman Mercado-Silva [21], Wendy Monk [22], Keigo Nakamura [23], George G. Ndiritu [24], Ralph Ogden [25], Michael Peat [26], Trefor B. Reynoldson [27], Blanca Rios-Touma [28], Pedro Segurado [8], and Adam G. Yates [29]

1 Department of Life Sciences, MARE-Marine and Environmental Sciences Centre, University of Coimbra, 3000-456 Coimbra, Portugal
2 Amnis Opes Institute, Corvallis, OR 97333, USA; hughes.bob@amnisopes.com
3 Department of Fisheries & Wildlife, Oregon State University, Corvallis, OR 97331, USA; kaufmann.phil@epa.gov
4 Laboratory of Ecology of Benthos, Department of Genetic, Ecology and Evolution, Institute of Biological Sciences, Federal University of Minas Gerais, Avenida Antônio Carlos 6627, CEP 31270-901 Belo Horizonte, MG, Brazil; callistom@ufmg.br
5 Centre for Applied Water Science, Institute for Applied Ecology, University of Canberra, 2601Canberra, Australia; sue.nichols@canberra.edu.au (S.J.N.); fiona.dyer@canberra.edu.au (F.J.D.)
6 Unilever Centre for Environmental Water Quality, Institute for Water Research, Rhodes University, P.O. Box 94, Grahamstown 6140, South Africa; odume.nelson@gmail.com
7 MARE – Marine and Environmental Sciences Centre, University of Évora, 7000-812 Évora, Portugal; bsquintella@fc.ul.pt
8 Department of Animal Biology, Faculty of Sciences of the University of Lisbon, Campo Grande, 1749-016 Lisboa, Portugal
9 Department of Zoology, School of Natural Sciences, Trinity Centre for the Environment, Trinity College Dublin, The University of Dublin, College Green, Dublin 2, Ireland; m.kuemmerlen@tcd.ie
10 Centro de Estudos Florestais, Instituto Superior de Agronomia, Universidade de Lisboa, Tapada da Ajuda, 1349-017 Lisboa, Portugal; fraguiar@isa.ulisboa.pt (F.C.A.); psegurado@isa.ulisboa.pt (F.S.)
11 Department of Biology and GeoBioTec - GeoBioSciences, GeoTechnologies and GeoEngineering Research Centre, University of Aveiro, Campus de Santiago, 3810-193 Aveiro, Portugal; salmeida@ua.pt
12 Mexican Institute of Water Technology, Bioindicators Laboratory, Jiutepec Morelos 62550, Mexico; palonso@tlaloc.imta.mx
13 Department of Animal and Environmental Biology (Applied Hydrobiology Unit), Federal University of Technology, P.M.B. 65 Minna, Nigeria; francisarimoro@gmail.com
14 School of Biologcal Sciences, University of Canterbury, 8140 Christchurch, New Zealand; jon.harding@canterbury.ac.nz
15 Department of Civil Engineering, Daejin University, Hoguk-ro, Pocheon-Si, Gyeonggi-Do 1007, Korea; drjang@daejin.ac.kr
16 Pacific Ecological Systems Division, Center for Public Health and Environmental Assessment, Office of Research and Development, U.S. Environmental Protection Agency, Corvallis, OR 97333, USA;
17 Korea Institute of Civil Engineering and Building Technology (KICT), 283 Goyangdaero, Ilsanseo-Gu, Goyang-Si, Gyeonggi-Do 10223, Korea; samhee.lee@kict.re.kr
18 Key Laboratory of Yangtze River Water Environment, Ministry of Education of China, Tongji University, Shanghai 200092, China; leejianhua@tongji.edu.cn
19 Department of Geography, Geomorphology and Water Resources Laboratory, Institute of Geosciences, Federal University of Minas Gerais, Avenida Antônio Carlos 6627, CEP 31270-901 Belo Horizonte, MG, Brazil; diegorm@ufmg.br
20 MED – Instituto Mediterrâneo para a Agricultura, Ambiente e Desenvolvimento, LabOr – Laboratório de Ornitologia, Universidade de Évora, Polo da Mitra, 7002-774 Évora, Portugal; aimendes@uevora.pt

[22] Centro de Investigación en Biodiversidad y Conservacíon, Universidad Autónoma del Estado de Morelos, Cuernavaca, 62209 Morelos, Mexico; norman.mercado@uaem.mx
[22] Environment and Climate Change Canada and, Canadian Rivers Institute, Faculty of Forestry and Environmental Management, University of New Brunswick, Fredericton, NB E3B 5A3, Canada; wendy.monk@canada.ca
[23] Water Environment Research Group, Public Works Research Institute, 1-6 Minamihara, Tsukuba 305-8516, Japan; nakamura-k573bs@pwri.go.jp
[24] School of Natural Resources and Environmental Studies, Karatina University, P. O. Box 1957, 10101 Karatina, Kenya; gatereg@yahoo.com
[25] Environment, Planning and Sustainable Development Directorate, 2601 Canberra, Australia; ralph.ogden@act.gov.au
[26] Wetlands, Policy and Northern Water Use Branch, Commonwealth Environmental Water Office, 2601 Canberra, Australia; michael.peat@awe.gov.au
[27] Acadia University, Canada Creek, Wolfville, NS B0P 1V0, Canada; trefor.reynoldson@gmail.com
[28] Grupo de Investigación en Biodiversidad, Medio Ambiente y Salud (BIOMAS), Facultad de Ingenierías y Ciencias Aplicadas, Ingeniería Ambiental, Universidad de Las Américas, Vía Nayón S/N, 170503 Quito, Ecuador; blanca.rios@udla.edu.ec
[29] Department of Geography, Western University and Canadian Rivers Institute, London, ON N6A 5C2, Canada; adam.yates@uwo.edu
* Correspondence: mjf@ci.uc.pt

Supplementar Material

Table S1. Examples of ecological monitoring networks/programs of rivers and streams implemented in the World (based on the countries considered by this study) by official authorities or “seed programs” by research teams (where no official program is available).

**Continent/ country**	**Scale** (national/state/regional /catchment/other)	**Law addressing the ecological assessment (if any)**	**Biological elements monitored**
**AFRICA**			
South Africa	National	National Water Act	Fish, riparian vegetation and invertebrates
South Africa, Namibia, Botswana and Lesotho	Catchment - transboundary Orange-Senqu River basin	Orange-Senqu River Commission	Fish, riparian vegetation and invertebrates
Lesotho		Lesotho Highlands Water Project	Invertebrates, fish and riparian vegetation
**ASIA**			
China		Law of the People’s Republic of China on Water and Soil Conservation & Opinions of the State Council on the Implementation of the Strictest Water Resources Management System (2012)	Fish, aquatic mammals, benthic animals, epiphytic algae, phytoplankton, aquatic vascular plants, waterside vegetation, beach vegetation, benthic animals, amphibians, reptiles, wetland birds, Indigenous, rare, endangered and endemic species
Japan	National	National Census on the River Environment	Fish, benthic invertebrates, plants, birds
South Korea	National	Water Quality and Aquatic Ecosystem Conservation Act – from 2008<br>Ministry of Environment's Water Environment Conservation Act – from 2018	Diatoms, invertebrates, riparian vegetation
Singapore		Public Utilities Board of the Singapore Government	Invertebrates
**EUROPE**			
European Union (27 MS + UK and Norway)	European/National	European Water Framework Directive & national legislations	Invertebrates, diatoms, macrophytes and fish are regularly monitored each 3 years to determine the ecological status of all water bodies
**CENTRAL & SOUTH AMERICA**			
Equador	National – but only for environmental impact assessment	Ecuadorian normative for Mining Impact assessment	Invertebrates
Costa Rica	National	Costa Rica, 2007; Política Nacional de Áreas de Protección de Ríos Quebradas, Arroyos y Nacientes (2020)	Invertebrates

Brazil	National law	CONAMA Resolution No 357 (Brasil 2005)	The law considers biological elements but is not implemented
Colombia		Colombia 2018	The law considers the use of biological elements but is not implemented
**OCEANIA**			
Australia – Vitoria, Queensland, Australian Capital Territory	State:<br>Victoria (VEFMAP & WetMAP)	None	VEFMAP: fish and riparian vegetation<br>WetMAP: fish, birds, vegetation and frogs
Australia	State: Australian Capital Territory (Catchment Health indicator program)	None	Macroinvertebrates, water quality and riparian condition
New Zealand	Catchment - Australia's Murray Darling Basin	The Water Act (2007)	Fish communities, groundcover vegetation diversity and stream metabolism, waterbirds and less frequently, frogs and tree condition
NORTH AMERICA			
USA	National and some States (California, Iowa, Maryland, Ohio, Oregon)	Clean Water Act	Invertebrates and fish
Canada	Province		Invertebrates; fish to a lesser extent
Mexico	Some catchments (i.e., Ayuguila, Armería, Pánuco, Sonora, Balsas and Bravo rivers)		Invertebrates; fish to a lesser extent

Table S2. Examples of rehabilitation of rivers around the world aiming the improvement of the biological assemblages.

**Case study/ country**	**Scale**	**Aims /Targets for restoration**	**What triggered the program?** (legislation & problems)	**Pre/Post ecological monitorin g**	**Success**	**References**
**AFRICA**						

Working for Water Programme/ South Africa	National	Reduction in the density of terrestrial, invasive alien plants on river catchments, by 22% per annum. Improve stream flow and general river ecological condition and function, job creation, livelihoods diversification and environmental education and awareness raising.	National Water Act (Act No. 36 of 1998) National Environmental Management Act (NEMA) Act No. 107 of 1998); Conservation of Agricultural Resources Act	Yes	The programme has been largely successful, improving stream flow, and thus biodiversity and has generated thousands of jobs.	**410** https://www.environment.gov.za/projectsprogrammes/wfw#aims
The Tsitsa River Project/South Africa	National Water Act and National Environmental Management Act	Sustainable restoration and improvement of land, water resources and livelihoods diversification. Restore degraded land, reduce siltation and improve livelihoods	National Water Act and National Environmental Management Act /Planned construction of dams on the Tsitsa River, fear of siltation due to degraded land and erosivity	Yes	The project is still on-going; it was successful so far due to the involvement of communities, diverse practitioners and academic knowledge systems.	**411**
**ASIA**						
Taihu Basin/China	22 major rivers (120 000	Improve water quality and ecological	The Taihu Basin	Yes, monitoring of blue-	Water quality indicators are steadily	**412**

	km^2) and 1 large catchment (233800 ha)	health, enhance connectivity of rivers and lakes. Severe cyanobacteria outbreak.	Management Regulations	green algae and water resources	decreasing, and the water ecology is improving	
Yangtze River /China	11 province and province-level municipalities	Priority protection species and are of the Yangtze River and stop its over development. Species and biological resource protection, protection and restoration of habitat and wetlands	Outline of the Development Plan for Yangtze River Economic Belt, Action plan of the Yangtze River Protection and restoration campaign	Yes, a water quality monitoring, phytoplankton, zooplankton, living algae, fry and fish is gradually carried out	Still ongoing	**413**
Itachi River/Japan	3km in Yokohama city	Flood control and Restoration of highly urbanized river	River Law /Flood control and People's demand for natural river	Yes, monitoring of plants and river geomorphology	Successful; Civil Engineering Design Prize 2016	**80**
Tama River/Japan	Two sites within 1km	Conservation of endangered plant species (Aster Kantonensis) and restoration of gravel river bed	River Law/ Incised river channel and endangered species	Yes, monitoring of plants and river geomorphology	Successful	**80**
Kushiro River (Mire)/Japan	2.4 km meander river restored	Restore in-stream habitat for native fish, invertebrates	River Law and Law for the Promotion of Nature	Yes	Successful, not only the natural landscape of a meandering	**332**

		floodplain vegetation by increasing flooding frequency and raising the groundwater table; reduce sediment and nutrient loads in the wetland areas restore a river–floodplain landscape	Restoration/ Degradation of Kushiro mire: sediment increments and drying wetland		river but also its function	
Kamisaigo River/Japan	Ca. 1km in urban area	Flood control Restoration of river environment and relation between river and residents	River Law	Yes, monitoring of fish fauna, physical environment, and vegetation	Successful, Civil Engineering Design Prize 2016	**333**
Anyangchen River - Korea	32km in main stream and 4 tributaries within Anyang city	Improving water environment, ecosystem and flood mitigation in urban streams. Promote harmony between Flood Management and Ecological Environment	Water Environment Conservation Act. Improve the quality of life of urban residents (Yangjaecheon river success story was a catalyst)	Yes, monitoring water quality, geomorphology, flora and fauna, so far	Successful - first award for best practice of river restoration (a typical case of Korea's restoration of urban stream)	**414**
Cheonggyecheon River (started in 2001–completed in 2010) -Korea	5.8km in Seoul city	Improving water environment, ecosystem and safety for	Local government Ordinace, River Act,	Yes, monitoring water quality, air pollution	Successful - removed roads from streams used as motorways	https://www.ser-rrc.org/project/south-korea-restoration-of-

		flood in urban streams. To promote harmony between Flood Management and Ecological Environment	Water Environment Conservation Act. Improve the quality of life of urban residents	along waterfront, flora and fauna, so far	and restored streams along with the improvement of urban landscapes	the-cheonggyecheon-river-in-downtown-seoul/
Yangjaecheon Stream Restoration (started in 1995 – complete) – Korea	3.5km in Gangnam district of Seoul City	Improving water environment, ecosystem and setting up rest facilities in urban streams.To restoring stream that citizens have shunned as near stream through a clean and natural appearance	Funding from a private company. Ecological restoration and urban river environmental park project	Yes, monitoring water quality, geomorphology, flora and fauna, landscape	Successful- First river restoration case in Korea (It triggered restoration of rivers nationwide in Korea)	http://www.korea.net/NewsFocus/Travel/view?articleId=142266
**EUROPE**						
Mondego river/Portugal	River stretch of 65 km	Improve longitudinal connectivity for migratory fish (diadromous); several fish passages were implemented	Endangered and economically important fish species	Yes, annual monitoring to assess the abundance of larvae or juveniles of some species (electric fishing); Biotelemetry to evaluate	Yes. Recovery of habitat for all target fish species; the larvae of sea lamprey (Petromyzon marinus) increased up to 100x when compared with the monitoring data obtained before the	**363**<br><br>**364**

				the fish passage efficiency; visual census conducted at the fish passage monitoring window to count the anadromo us spawners that enter every year to spawn	construction of the fish passages	
River Vienne/France	River stretch of 15 km	Improve the connectivity for migratory fish - removal a dam due to its numerous effects on river continuity and its low level of economic benefit /	Absence of migratory fish upstream the dam; poor effect of fish-passes. Long-term management plan for the River Loire)	Yes, monitoring of migratory fish	Re-activation of sediment transport, for anadromous fish, with evidence of recolonization and increasing reproduction reconnection of 35k of river	https://professionnels.ofb.fr/sites/default/files/pdf/rex_r1_vienne_vbatGB.pdf
Isar River/Germany	River stretch of 8 km	Improving resilience to flooding events, restoring the natural conditions of the riverine landscape, enhancing the recreational value. Re-widening of	Flood protection standards and need for recreational spaces	The presence of several biological groups was assessed: fish, amphibians, birds, terrestrial invertebrates	Stronger dynamics in river morphology; wide acceptance of the improved recreational space; increased biodiversity	https://www.wwa-m.bayern.de/fluesse_seen/massnahmen/isarplan/doc/isar_river.pdf

		the channelized river; replacement of cross-river sills by rough ground ramps; construction new gravel beds and new floodplains; upgrade of waste-water treatment plants				
Sokołówka River/ Lodz/Poland	Urban river section	Improved storm-water management, increased water retention, and better water quality supporting higher biodiversity and improvement of quality of life/ encourages society healthy lifestyles, attracts business, and become resilient to global climate change	Floods in the city during storms are result of high proportion of impermeable surfaces and channelization/Degradation of the freshwater habitats/Poor water and ecological quality	Yes, assessment of fish assemblages and RNA/DNA ratio in fish tissues.	Yes, in terms of stormwater retention; aesthetic value; environmental quality in the urban space; no clear indications for aquatic biological elements	https://climate-adapt.eea.europa.eu/metadata/case-studies/urban-river-restoration-a-sustainable-strategy-for-storm-water-management-in-lodz-poland/#adapt_options_anchor; http://www.switchurbanwater.eu/outputs/pdfs/W6-2_CLOD_RPT_SWITCH_City_Paper_-_Lodz.pdf
Bernesga River/ Duero river basin/Spain	Dam area	Removal of a dam and all mechanisms	Spanish National Strategy of	Yes, assessment of fish and	Yes, the connectivity of the river was	https://damremoval.eu/portfolio/la-

		associated to gates and all concrete material; reestablishment of natural connectivity, migration of fish and passage for macroinvertebrates and other species	River Restoration; accumulation of sediment above dam and deficit downstream; barrier for fish	macroinvertebrates (before and one-year data after the dam removal)	restored; sediments were naturally transported downstream; macroinvertebrate indices improved downstream	gotera-dam-spain/
**CENTRAL & SOUTH AMERICA**						
Upper Guayllabamba River Basin	Small tributary stretches: Ortega and Shanshayacu streams	Rehabilitation of urban streams due to garbage accumulation, health hazards and recover of green spaces for neighborhoods/Riparian clean up, riparian vegetation recovery, bank stabilization	Unsafe conditions of stream riparian areas, and awareness from neighbors.	No	Only visual riparian recovery	**338**
La Vieja River Basin, Colombia	Basin wide management, with protection of riparian corridors.	Restore riparian and stream quality from the effect of extensive cattle	A basin scale project to switch extensive agriculture to silvopastoral systems	Riparian, physical habitat, macroinvertebrate community, water quality	Yes	**339**
Nutrias stream, Argenitna	Reach scale	Recover from intensive/extensive cattle	A research program to address	Riparian, habitat, bank and	Yes	**415**

			passive restoration intervention in Pampean Streams	fish communit y		
Pichis River, Perú	River basin	Riparian restoration for fish community recovery	Decreases in fisheries and disappearan ce of target species	Yes	Yes, fish re-appearance due to riparian forest recovery	**341**
Urban streams of Belo Horizonte city, catchment of Rio das Velhas	Streams	Improve water quality, aesthetic value, human health	Water Master Plan (Belo Horizonte 1999, 2012)/ Pollution and degradation of the urban streams causing risks to human health and contributing to poor living conditions in the surrounding s	Yes, 10 years of follow-up monitoring (Recurb project)	Improvement of water quality, species richness, composition and assemblage structure of benthic invertebrate communities, and appearance of new sensitive taxa	**121,370,368**
**OCEANIA**						
MDB Basin Plan/Australia	River basin (>1 M km2)	To bring the basin back to a healthier and sustainable level, while continuing to support farming and other industries for	Multiple large-scale condition assessment programs showing that the basins rivers were in poor ecological	Yes (LTIM & MER)	Some site specific successes: supporting waterbird breeding, increasing productivity, supporting fish breeding.	**332,313, 314 416,321, 417**

		the benefit of the Australian community/ Improvement of riparian vegetation, fish community, bird habitat, river banks, flow	condition and drought.			
Murrumbidgee River Restoration Program/Australi a	River basin (>80,000 km2)	Improve water quality	NHT/Poor water quality	No (ad-hoc)	Unknown	**418**
Victorian environmental flows program/ Australia/	River basin (size)	Improve ecological condition	Flora and fauna bulk entitlement	Yes	Somewhat	**419,420,421**
Macquarie Perch Action Plan (for Cotter River and other ACT rivers)/Australia	Several catchments in ACT region	Enhance the long-term viability of populations	Nature Conservatio n Act 2014/Endan gered species status	Yes	Yes, although their status is still endangered	**398**
ACT Water Strategy (for all ACT waterways)/Aust ralia	Several catchments in ACT region	Maintain or improve the quality of water across all ACT managed sub-catchments Healthy catchments and waterbodies	Water Resources Act 2007, Environment protection Act 1997 & others/ Reform programs in biodiversity conservation and water quality and quantity management occurring	Yes	Examples of both success and failure	**399,422**

			Australia-wide			
Northern basin connectivity/Australia	Multiple catchments including Border Rivers, Gwydir, Macquarie and Barwon-Darling	To improve connectivity between catchments and reduce the number of cease to flow days in the Barwon-Darling/Fish condition and local movement	Commonwealth and state laws/ Disturbance (drought combined with legislative changes that impacted on small and medium in channel flows)	Yes	Leading to changes in water sharing arrangements. It could take decades to see the ecological benefits.	**319**
Project River Recovery (Waitaki River)/New Zealand	1 large catchment (35,000 ha)	Enhance habitat for Nationally threatened riverine birds/Riparian willow removal	Compensation for hydro development /Threatened species	Yes. Bird reproduction, predator & weed control monitoring	Increased bird habitat, some improvement in bird numbers	**423**
Zealandia (Karori Sanctuary)/New Zealand	1 urban catchment	Predator control	Sanctuary for threatened species	Fish monitoring	Invasive fish species removed	https://www.visitzealandia.com/Portals/0/Resources/20200128%20Sanctuary%20to%20Sea%20Strategy_Final%20Version.pdf?ver=2020-02-17-092719-310&timestamp=1581884864923
Upper Silverstream Creek/New Zealand	1 km of small, 1st order stream	Improve water quality and ecological health/Riparian planting,	Landowner initiative	Yes, water quality, benthic invertebrates,	Some improvement in ecological health	https://www.canterbury.ac.nz/science/schools-and-departments/

		sediment & macrophyte control		macrophytes, decomposition, fish		biological-sciences/research/ferg/carex/
**NORTH AMERICA**						
Willamette Basin/USA	River basin	Improve water quality, fish assemblages Salmon	Clean Water Act (CWA)/ Water pollution	Yes	Somewhat	**424**
Scioto River Mature/USA	Mainstem	Improve Water quality, fish & macroinvertebrate assemblages	CWA/Water polllution	Yes,	Mostly	**378**
Kissimmee River/USA	Mainstem	Naturalize/reconfigure the channel; improve water quality, channel complexity, waterfowl	Endangered Species Act (ESA) /Ecological degradation	Yes	Somewhat	**425**
Elwha River/USA	Mainstem	Promote Salmon passage/dam removal, improve fish assemblages	Endangered Species Act (ESA)/Migration barriers	Yes	Mostly	**426**
Bow River/Canada	mainstem	Sport Fishery/Tertiary sewage treatment and P removal	Excessive macrophyte growth	Yes	Yes	**427**
Sackville River/Canada	Mainstem and tributaries	Habitat and passage	Atlantic Salmon Fishery/Habitat loss and barriers	No		**428**

Ayuquila River Complete Mexico	Mainstem (partial)	Improve water quality	NOM-001-SEMARNAT-1996/ Water pollution/ Fish mortality	Yes	Yes, previously and ongoing	**293**
Rio Magdalena Early Mexico	Basin	Water quality Improvement and conservation	Water pollution	No	No information yet	**429**,**430**
Teuchitlan Stream Early (Ongoing) Mexico	Basin	Endangered species reintroduction /Water quality, habitat	Species loss	Yes	Yes, previously and ongoing	**431**

80. Nakamura, K.; Tockner, K.; Amano, K. River and Wetland Restoration: Lessons from Japan. Bioscience 2006, 56, 419–429.

121. Feio, M.J.; Ferreira, W.R.; Macedo, D.R., Eller, A.P.; Alves, C.B.M.; França, J.S.; Callisto, M. Defining and testing targets for the recovery of tropical streams based on macroinvertebrate communities and abiotic conditions. River Res. Appl. 2015, 31, 70–84.

293. Mercado-Silva, N.; Lyons, J.D.; Maldonado, G.S.; Nava, M.M. Validation of a fish-based index of biotic integrity for streams and rivers of central Mexico. Rev. Fish Biol. Fish. 2002, 12, 179–191.

313. Norris, R.H.; Prosser, I.; Young, B.; Liston, P.; Bauer, N.; Davies, N.: Dyer, F.; Linke, S.; Thoms, M. The Assessment of River Condition (ARC). An audit of the ecological condition of Australian Rivers. In Canberra, Australia. Available online http://iae.canberra.edu.au/reprints/2001_Norris_etal_The_assessment_of_river_condition.pdf (accessed on 23 March 2015).

314. Norris, R.H.; Linke, S.; Prosser, I.; Young, W.J.; Liston, P.; Bauer, N.; Sloane, N.; Dyer, F.; Thoms, M. Very-broad-scale as-sessment of human impacts on river condition. Freshw. Biol. 2007, 52, 959–976.

319. DAWE—Delivering environmental works and measures for the northern Basin. Department of Agriculture, Water and En-vironment 2020. Available online:

https://www.agriculture.gov.au/water/mdb/basin-plan/commitments/environmental-works-measures (accessed on 30 June 2020).

321. Stewardson, M.J.; Guarino, F. Basin-scale environmental water delivery in the Murray–Darling, Australia: A hydrological perspective. Freshw. Biol. 2018, 63, 969–985.

332. Nakamura, F.; Ishiyama, N.; Sueyoshi, M.; Negishi, J.N.; Akasaka, T. The significance of meander restoration for the hydro-geomorphology and recovery of wetland organisms in the Kushiro River, a lowland river in Japan (in Japanese with English abstract). Restor. Ecol. 2014, 22, 544–554.

333. Hayashi, H.; Shimatani, Y.; Hattori, M. A Practice of Stream Restoration Project in Urban Area and Its Evaluation. In Pro-ceedings of 11th International Symposium on Ecohydraulics, Melbourne, Australia, 7–12 February 2016.

338. Da Cruz e Sousa, R.C.; Ríos-Touma, B. Stream restoration in Andean cities: Learning from contrasting restoration approaches. Urban Ecosyst. 2018, 21, 281–290.

339. Pedraza, G.; Giraldo, L.; Chará, J. Effect of Restoration of Riparian Corridors on the Biotic and Abiotic Characteristics of Streams in Cattle Ranching Areas of La Vieja River Catchment in Colombia. Zootec. Trop. 2008, 26, 179–182.

341. Aldave, M.; Castro, E.; Summers, P.; Tipula, P. Restoration of Riverine Forests: Contributions for Fisheries Management in the Pichis River Watershed of the Selva Central Region of Peru BT. In Social-Ecological Systems of Latin America: Complexities and Challenges; Delgado, L.E., Marín, V.H., Eds.; Springer International Publishing: Cham, Switzerland, 2019; pp. 367–387, ISBN 978-3-030-28452-7.

363. Pereira, E.; Quintella, B.R.; Mateus, C.S.; Alexandre, C.M.; Belo, A.F.; Telhado, A.; Quadrado, M.F.; Almeida, P.R. Performance of a Vertical-Slot Fish Pass for the Sea Lamprey Petromyzon marinus L. and Habitat Recolonization. River Res. Appl. 2017, 33, 16–26.

364. Moser, M.L.; Almeida, P.R.; Kin, J.J.; Pereira, E. Passage and freshwater habitat requirements of anadromous lampreys: Con-siderations for conservation and control. J. Great Lakes Res. 2020, doi:10.1016/j.jglr.2020.07.011.

368. Hughes, R.M.; Gammon, J.R. Longitudinal changes in fish assemblages and water quality in the Willamette River, Oregon. Trans. Am. Fish. Soc. 1987, 116, 196–209.

370. Kauffman, J.B.; Beschta, R.L.; Otting, N.; Lytjen, D. An ecological perspective of riparian and stream restoration in the west-ern United States. Fisheries 1997, 22, 12–24.

378. Yoder, C.O.; Rankin, E.T.; Gordon, V.L.; Hersha, L.E.; Boucher, C.E. Degradation and recovery of the Scioto River (Ohio-USA) fish assemblage from presettlement to present-day conditions. In From Catastrophe to Recovery: Stories of Fishery Management Success; Krueger, C.C., Taylor, W.W., Youn, S., Eds.; American Fisheries Society: Bethesda, MD, USA, 2019; pp. 233–265.

398. ACT Government. Macquarie Perch Macquaria australasica Action Plan. Commonwealth of Australia, 2018. Available online: https://www.environment.gov.au/system/files/resources/bdee49ef-45da-4eb7-b548-bcfce460a21b/files/recovery-plan-macquarie-perch-2018.pdf (accessed on 3 September 2020).

399. ACT Government. Water Resources Environmental Flow Guidelines 2019; Disallowable Instrument DI2019—37 Made under the Water Resources Act 2007, s12 (Environmental Flow Guidelines); ACT Government: Canberra, Australia, 2019.

410. Binns, J.A.; Illgner, P.M.; Nel, E.L. Water shortage, deforestation and development: South Africa's working for water pro-gramme. Land Degrad. Dev. 2001, 12, 341–355.

411. Bannatyne, L.J.; Rowntree, K.M.; van der Waal, B.W., Nyamela, N. Design and implementation of a citizen technician-based suspended sediment monitoring network: Lessons from the Tsitsa River catchment, South Africa. Water 2017, 43, 365–377.

412. Hu, H.; Tan, J. Review and Reflection on comprehensive treatment of water environment in Taihu Basin of Jiangsu Province. Chin. Consult. Eng. 2019, 3, 92–96.

413. Su, L.; Liu, Y. Assessment on the Progress of the Institutional Improvement of the Yangtze River Protection. Environ. Protect. 2019, 47, 14–21.

414. Lee, S.H.; Choi, J.K. A Study on the Application and Assessment of Urban River Restoration in the Anyang River. J. Korean Soc. Environ. Res. Technol. 2007, 10, 1–8.

415. Giorgi, A.; Rosso, J.J.; Zunino, E. Efectos de la exclusión de ganado sobre la calidad ambiental de un arroyo pampeano. Biol. Acuát. 2014, 30, 133–140.

416. Watts, R.J.; Dyer, F.; Frazier, P.; Gawne, B.; Marsh, P.; Ryder, D.S.; Southwell, M.; Wassens, S.M.; Webb, J.A.M.; Ye, Q.F. Learning from concurrent adaptive management in multiple catchments within a large environmental flows program in Australia. River Res. Appl. 2020, 36, 668–680.

417. Hale, J.; Bond, N.; Brooks, S.; Capon, S.; Grace, M.; Guarino, F.; James, C.; King, A.; McPhan, L.; Mynott, J.; et al. Murray–Darling Basin Long Term Intervention Monitoring Project—Basin Synthesis Report; Report prepared for the Agriculture, Water and the Environment, Commonwealth Environmental Water Office by La Trobe University, Centre for Freshwater Ecosystems; CFE Publication: Melbourne, Australia, 2020; p. 59.

418. Higgisson, W.P.; Downey, P.O.; Dyer, F.J. Changes in Vegetation and Geomorphological Condition 10 Years after Riparian Restoration. Water 2019, 11, 1252, doi:10.3390/w11061252.

419. Koster, W.M.; Amtstaetter, A.; Dawson, D.; Coleman, R.A.; Hale, R. Environmental influences on the juvenile migration of the threatened amphidromous Australian grayling (Prototroctes maraena). Mar. Freshw. Res. 2020, doi:10.1071/MF20039.

420. Tonkin, Z.; Yen, J.; Lyon, J.; Kitchingman, A.; Koehn, J.D.; Koster, W.M.; Lieschke, J.; Raymond, S.; Sharley, J.; Stuart, I.; et al. Linking flow attributes to recruitment to inform water management for an Australian freshwater fish with an equilibrium life-history strategy. Sci. Total Environ. 2020, doi:10.1016/j.scitotenv.2020.141863.

421. Vivian, L.; Greet, J.; Jones, C.S. Responses of grasses to experimental submergence in summer: Implications for the manage-ment of unseasonal flows in regulated rivers. Aquat. Ecol. 2020, doi:10.1007/s10452-020-09788-4.

422. ACT Government. ACT Water Strategy Report Card for Implementation Plan One (2014–18); ACT Government: Canberra, Aus-tralia, 2017.

423. Caruso, B.S. Effectiveness of braided, gravel-bed river restoration in the Upper Waitaki Basin, New Zealand. River Res. Appl. 2006, 22, 905–922.

424. Hughes, R.M.; Bangs, B.L.; Gregory, S.V.; Scheerer, P.D.; Wildman, R.C.; Ziller, J.S. Recovery of Willamette River fish assem-blages: Successes & remaining threats. In From Catastrophe to Recovery: Stories of Fishery Management Success; Krueger, C.C., Taylor, W.W., Youn, S., Eds.; American Fisheries Society: Bethesda, MD, USA, 2019; pp. 157–184.

425. Cheek, M.D.; Williams, G.E.; Bousquin, S.G.; Colee, J.; Melvin, S.L. Interim response of wading birds (Pelecaniformes & Ciconifformes) and waterfowl (Anseriformes) to the Kissimmee River restoration Project, Florida, USA. Restor. Ecol. 2014, 22, 426–434.

426. Duda, J.J.; Anderson, J.H.; Beirne, M.; Brenkman, S.; Crain, P.; Mahan, J.; McHenry, M.; Pess, G.; Peters, R.; Winter, B. Com-plexities, context, and new Information about the Elwha River. Front. Ecol. Environ. 2019, 17, 10–11.

427. CPP-Hutchinson. Bow River Phosphorus Management Plan. Water Quality Data Analysis. Alberta Environment and Sustainable Development 2013. Available online: https://open.alberta.ca/dataset/d1557a73-7cf4-4e8f-b5b9-9ce40ae9f00f/resource/1dd9e8b2-0032-4afd-bd7a-fe24e90df377/download/2014-bow-river-phosphorus-management-plan-brpmp-water-quality-2014-06-10.pdf (accessed on 15 June 2020).

428. Sackville Rivers Association. River Restoration Final Report 2017; Sackville Rivers Association: Middle Sackville, NS, Canada, 2017; p. 19.

429. Zamora Saenz, I., Mazari Hiriart, M., Almeida Leñero, L. Sistema de indicadores para la recuperación de ríos urbanos. El caso del río Magdalena, Ciudad de México. Acta Univ. 2017, 27, 53–65.

430. Monsivais Montoliu, B. Programa de Rescate Integral del Río Magdalena en México, D.F. Master's Thesis, Instituto de Sos-tenibilidad, Universidad Politécnica de Cataluña, Barcelona, Spain, 2014.

431. Domínguez-Domínguez, O.; Morales, R.H.; Nava, M.M.; Diego, Y.H.; Venegas, D.T.; Jiménez, A.L.E.; Vázquez, L.H.E.; García, G. Progress in the reintroduction program of the tequila splitfin in the springs of Teuchitlán, Jalisco, Mexico. In Global Rein-troduction Perspectives: Case Studies from around the Globe; Soorae, P.S., Ed.; IUCN SSC Reintroduction Specialist Group and Abu Dhabi, United Arab Emirates; Environment Agency: Gland, Switzerland, 2018.
